# Physiological and transcriptomic insights into the molecular mechanisms of salt stress adaptation in *Gardenia jasminoides*

**DOI:** 10.1186/s12870-025-08042-z

**Published:** 2026-01-02

**Authors:** Wenhui Li, Ziyang Zhang, Dexing Chen, Xin He, Ye Huang, Xuewei Zhao, Weihong Sun, Zhong-Jian Liu, Shuang-Quan Zou

**Affiliations:** 1 College of Forestry, Fujian Agriculture and Forestry University, Fuzhou, 350002 China; 2 Fujian Colleges and Universities Engineering Research Institute of Conservation and Utilization of Natural Bioresources, Fujian Agriculture and Forestry University, Fuzhou, 350002 China; 3 China Strait Horticulture Technology Innovation Hub, College of College of Landscape Architecture and Art, Fujian Agriculture and Forestry University, Fuzhou, 350002 China; 4 College of Biology and Agriculture Technology, Zunyi Normal College, Zunyi, Guizhou 563000 China

**Keywords:** *Gardenia jasminoides*, Salt stress, Antioxidant defense, RNA-seq analysis, Reactive oxygen species (ROS)

## Abstract

**Background:**

Salt stress is a major environmental factor limiting plant growth and productivity. Plants have developed diverse physiological and molecular strategies to adapt to saline conditions. *Gardenia jasminoides*, a dual-purpose plant with significant economic value in medicine and landscaping, exhibits notable salt tolerance, but the underlying molecular mechanisms remain largely unexplored. Our study aims to elucidate the physiological and molecular adaptive strategies of this plant to salt stress by integrating physiological measurements and transcriptomic analysis.

**Results:**

RNA-seq profiling of *G. jasminoides* leaves, which were subjected to four NaCl concentrations (0, 50, 100, and 200 mmol·L⁻¹) for 15 days, identified 3,883 differentially expressed genes (DEGs). These DEGs were mainly associated with calcium signaling, MAPK–WRKY cascades, antioxidant defense, and phytohormone regulation. Physiological analysis revealed that prolonged stress caused structural damage to the photosynthetic apparatus, evidenced by a significant decline in *F*_*v*_/*F*_*m*_ and an increase in *F*_*o*_ at Day 15. These changes mirrored transcriptional reprogramming: peroxidase (POD) activity and related genes were strongly induced, catalase (CAT) activity was repressed, and malondialdehyde (MDA) levels increased under severe stress. Osmotic adjustment displayed stress-dependent patterns, with proline accumulation peaking under moderate stress and soluble proteins under severe stress. Network analysis highlighted a hierarchical regulatory system, where calcium signaling and MAPK cascades coordinated antioxidant defense and ion homeostasis. Key salt-responsive regulators, including *CaM/CML*, *CDPK*, *WRKY22/29*, and glutathione S-transferase (*GST*) family members, were proposed as central nodes integrating Ca²⁺ influx, reactive oxygen species (ROS) production, MAPK activation, and abscisic acid (ABA) signaling.

**Conclusions:**

Our findings reveal a hierarchical regulatory network that fine-tunes ROS homeostasis, osmotic balance, and stress signaling in *G. jasminoides*. Salt-responsive regulators such as WRKY transcription factors and *GST* family members represent promising targets for functional studies and molecular breeding aimed at enhancing salt tolerance in woody plants for ecological restoration and agricultural production.

**Supplementary Information:**

The online version contains supplementary material available at 10.1186/s12870-025-08042-z.

## Background

Salt stress profoundly impairs plant growth and development by disrupting multiple physiological and biochemical processes [1, 2]. Globally, the area of saline-affected soils continues to expand due to climatic anomalies and anthropogenic activities [3]. Excess Na^+^ accumulation induces osmotic stress, ion toxicity, and elevated reactive oxygen species (ROS) production, ultimately causing cellular dysfunction or plant death [4–6]. Plants counteract these stresses through mechanisms including ion homeostasis regulation, osmotic adjustment, antioxidant activation, and phytohormone modulation [7, 8]. *Gardenia jasminoides* is an economically and ecologically important woody plant widely cultivated in subtropical regions [9, 10]. Unlike common ornamental species, *G. jasminoides* serves a distinct dual purpose. In traditional medicine, its fruit has been utilized for millennia to treat inflammation, hepatic disorders, and hypertension [9, 11]. Modern pharmacology confirms that its fruits are a primary source of geniposide and crocin, which are valuable natural pigments and medicinal compounds with hepatoprotective and antioxidant properties [9, 12]. Simultaneously, it is highly valued in the horticultural industry for its fragrant flowers and evergreen foliage [9, 13]. However, *G. jasminoides* is sensitive to salt stress, and soil salinization restricts its geographical distribution and cultivation. Salt stress not only inhibits vegetative growth, compromising its landscaping value, but also significantly reduces fruit yield and the accumulation of these bioactive secondary metabolites [14]. Therefore, elucidating the molecular mechanisms of salt tolerance in *G. jasminoides* is critical for breeding cultivars that can thrive in saline environments, thereby securing both its ecological function and economic productivity [11, 15]. However, the development of such stress-resilient cultivars is currently hindered by a knowledge gap. Despite extensive physiological studies on its salt tolerance, the molecular mechanisms underlying this capacity remain insufficiently understood [16]. Integration of physiological responses with transcriptomic profiling under salt stress is therefore essential to elucidate tolerance mechanisms and construct genomic networks that regulate adaptation, particularly through functional characterization of differentially expressed genes (DEGs) [17]. Physiological responses, such as alterations in antioxidant enzyme activities and osmolyte accumulation, suggest that *G. jasminoides* employs complex strategies under salt stress 01. Antioxidant enzymes including peroxidase (POD), superoxide dismutase (SOD), and catalase (CAT) are critical for detoxifying ROS [18, 19]. POD activity typically increases with stress intensity, whereas SOD activity fluctuates across stress stages, reflecting its regulatory complexity. POD participates in H₂O₂ scavenging and lignin biosynthesis, SOD catalyzes the dismutation of superoxide radicals to H₂O₂, and CAT decomposes H₂O₂ into water and oxygen [20, 21]. Malondialdehyde (MDA), a lipid peroxidation product, is a reliable indicator of oxidative membrane damage [22]. Osmolytes such as soluble proteins and proline also contribute to osmotic balance, protein stabilization, and ROS detoxification during stress [23, 24]. Soluble protein levels initially decline but recover, peaking under severe stress (SS), whereas proline accumulation follows a triphasic response before stabilization [25, 26]. These physiological features highlight the complexity of *G. jasminoides* adaptation to salinity, yet the molecular regulation underlying these processes remains unresolved.

To address this gap, we investigated salt tolerance in *G. jasminoides* through an integrative approach combining physiological and transcriptomic analyses. We specifically focused on leaf tissues, as they serve as the primary sites for photosynthesis and are highly susceptible to ionic toxicity and oxidative damage under salt stress [27, 28]. By linking antioxidant enzyme dynamics, osmolyte accumulation, and gene enrichment in stress-associated pathways, we sought to elucidate how calcium signaling, MAPK cascades, and hormone interactions coordinate adaptive responses [29–31]. This study advances the mechanistic understanding of salt adaptation in *G. jasminoides* and provides a basis for enhancing salt tolerance in woody plants.

## Materials and methods

### Plant materials

Clonal seedlings of *G. jasminoides* derived from cuttings were obtained from the Quanzhou Urban Forest Park Development Center (Quanzhou, China), and the species identity was authenticated by Prof. Shuang-Quan Zou (Fujian Agriculture and Forestry University). Six-month-old seedlings with uniform growth and no visible signs of disease or pests were transplanted into plastic pots (0.08 m height, 0.1 m diameter), with one plant per pot. Each pot was filled with 250 g of air-dried soil and placed on a tray. The soil was watered slowly to saturation. A total of 36 plants were prepared for the experiment. Plants were maintained under standard greenhouse conditions at Fujian Agriculture and Forestry University: 25 ± 2 °C/20 ± 2 °C (day/night), 60–70% relative humidity, and a 14 h light/10 h dark photoperiod with a light intensity of ~ 300 µmol·m⁻²·s⁻¹. Healthy seedlings showing consistent growth were selected for salt stress treatments. These cultivated materials did not require specific collection permits, and no voucher specimens were deposited. All experiments complied with institutional, national, and international guidelines for plant research.

### NaCl treatment and sampling

**Fig. 1:**
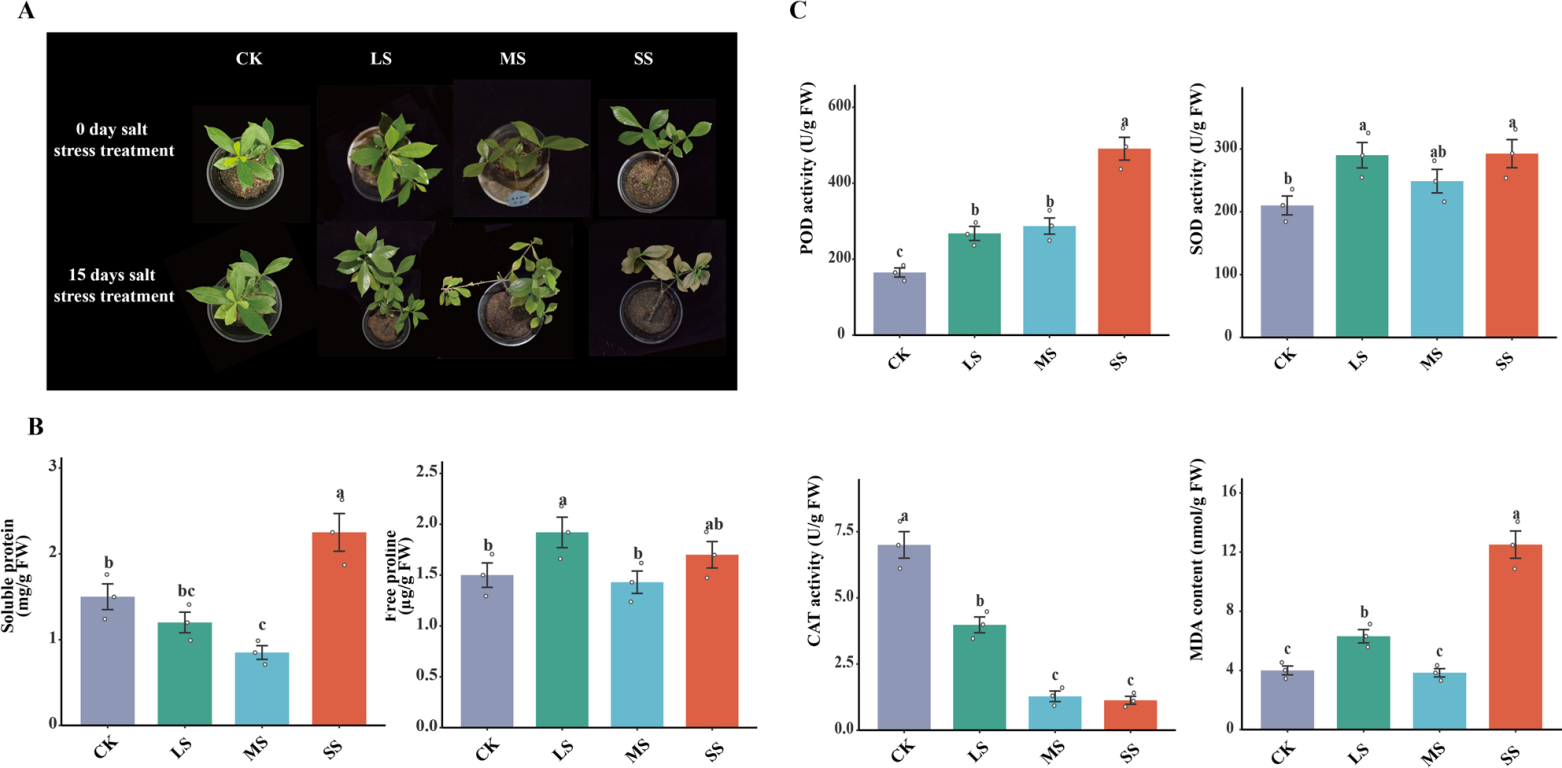
Effects of salt stress on the growth and physiological responses of *G. jasminoides.* **A** Phenotypic changes under different salt stress levels (CK: control; LS: low salt; MS: moderate salt; SS: severe salt) at 0 and 15 days. **B** Soluble protein and free proline contents in leaves. Error bars represent the standard error (SE) of the mean from three biological replicates. **C** Activities of antioxidant enzymes (POD, SOD, CAT) and MDA content after 15 days of salt treatment. Error bars represent the standard error (SE) of the mean from three biological replicates

Four NaCl treatments were established: control (CK, 0 NaCl), low salt stress (LS, 50 mmol·L⁻¹ NaCl), moderate salt stress (MS, 100 mmol·L⁻¹ NaCl), and severe salt stress (SS, 200 mmol·L⁻¹ NaCl) (Tab. S1). The experiment followed a randomized block design. Each treatment contained three biological replicates, and each replicate consisted of three individual plants, resulting in a total of 36 plants (4 treatments × 3 replicates × 3 plants). Beginning daily at 23:00, 50 mL of the corresponding NaCl solution was applied to each pot, with care taken to minimize salt loss. The treatments lasted for 15 days. Plant growth was photographed and recorded before and after treatment **(**Fig. 1A**)**.

For physiological and biochemical assays, leaves from each biological replicate were measured in triplicate (technical replicates). For RNA-seq analysis, to ensure sample representativeness and minimize individual variation, leaf tissues were harvested from all three plants within each biological replicate and pooled to form one composite sample. Consequently, three independent pooled biological replicates were sequenced for each treatment. The second fully expanded leaves from the shoot apex were harvested at the same time of day to reduce circadian effects, immediately frozen in liquid nitrogen, and stored at − 80 °C until analysis.

### Determination of antioxidant defense and osmotic adjustment

To ensure consistency, three plants were selected for each treatment, and the second fully expanded leaves from the shoot apex were tagged before stress induction. At each sampling time, tagged leaves were excised, gently cleaned with a moist soft cloth, and placed into pre-chilled cryovials. Samples were flash-frozen in liquid nitrogen and stored at − 80 °C until analysis. The following assays were conducted using standard protocols: CAT (EC 1.11.1.6) activity was determined by hydrogen peroxide consumption [32]; POD (EC 1.11.1.7) activity was measured using the guaiacol oxidation method [33]; SOD (EC 1.15.1.1) activity was evaluated by the nitroblue tetrazolium (NBT) photoreduction assay [33]; MDA content was assessed via thiobarbituric acid reactive substances (TBARS) assay [34]; soluble protein content was measured by Coomassie Brilliant Blue G-250 staining [35]; and proline concentration was determined using the acidic ninhydrin method [36].

### Measurement of chlorophyll fluorescence parameters

Chlorophyll fluorescence parameters were measured using a portable chlorophyll fluorometer (OS-30p^+^, Opti-Sciences, Hudson, NH, USA). To ensure complete dark adaptation of the photosystems, measurements were conducted between 21:00 and 24:00 on the sampling days. For each treatment, three healthy, fully expanded leaves from three distinct plants were tagged and measured. The instrument settings were configured with a saturating pulse intensity of 3000 µmol m^− 2^ s^− 1^ and a pulse duration of 1.5 s. Minimal fluorescence (*F*_*o*_) was determined using a weak modulated measuring light, followed by a saturating pulse to record maximal fluorescence (*F*_*m*_). The variable fluorescence (*F*_*v*_), maximum quantum yield of PSII (*F*_*v*_/*F*_*m*_), and potential photochemical activity (*F*_*v*_/*F*_*o*_) were calculated based on the following equations: *F*_*v*_ = *F*_*m*_ – *F*_*o*_, *F*_*v*_/*F*_*m*_ = (*F*_*m*_ – *F*_*o*_)/*F*_*m*_, and *F*_*v*_/*F*_*o*_ = (*F*_*m*_ – *F*_*o*_)/*F*_*o*_.

### Transcriptome sequencing and annotation

Total RNA was extracted from leaves using the FastPure Plant Total RNA Isolation Kit (Vazyme Biotech Co., Ltd., Nanjing, China). To ensure the reliability of the sequencing data, RNA quality was rigorously assessed. RNA degradation and contamination were monitored on 1% agarose gels, and purity was checked using a NanoDrop spectrophotometer (IMPLEN, CA, USA). RNA integrity and concentration were precisely quantified using an Agilent 2100 Bioanalyzer (Agilent Technologies, CA, USA) with the Agilent RNA 6000 Nano Kit. Key parameters, including the RNA Integrity Number (RIN), 28 S/18S ratio, and total concentration, were evaluated. Only RNA samples meeting the library construction requirements of the sequencing provider were selected for downstream analysis. Short-read RNA-seq was conducted on frozen leaf tissue by the Beijing Genomics Institution (BGI, China) using the DNBSEQ-T7 platform. Purified PCR products were subjected to paired-end sequencing according to standard protocols [37]. Quality control was performed with fastp, and reads were further screened using FaQCs when necessary [38]. Clean reads were aligned with HISAT2 v2.1.1 against the published *G. jasminoides* genome (Accession: GCA_013103745.1) [39, 40]. Transcripts were reconstructed using StringTie [41].

### Statistical analyses

For physiological and biochemical assays, data are presented as mean ± SE (*n* = 3 biological replicates per treatment). Normality and homoscedasticity were verified before analysis. Group means were compared using one-way analysis of variance (ANOVA) followed by Fisher’s Least Significant Difference (LSD) test (*P* < 0.05). Different lowercase letters above the bars or points indicate statistically significant differences between treatments. Statistical analyses and data visualization were performed using R software, utilizing the agricolae package [42] for LSD tests and the ggplot2 package for figure generation. Transcript abundance was quantified as fragments per kilobase of transcript per million mapped reads (FPKM) using RSEM [43]. Differential expression analysis was conducted with DESeq [44], applying the Benjamini–Hochberg method [45]to control false discovery rate (FDR). Genes with FDR < 0.05 and |log_2_FC| > 1 were considered significantly differentially expressed. Functional annotation was performed using Blast2GO [46] (E-value ≤ 1e⁻⁵) and KOBAS 3.0 [47] (hypergeometric test) to obtain Gene Ontology (GO) and Kyoto Encyclopedia of Genes and Genomes (KEGG) annotations.

### Real-Time PCR (qRT-PCR) validation for DEGs

Eight key DEGs were selected for validation. Primers for target genes and the reference gene were designed using Primer Premier 5, with specificity confirmed by Primer-BLAST (NCBI; accessed 20 May 2024). Primer details are listed in Table S2. Amplification efficiency, determined from standard curves, ranged from 90% to 110%. Specificity was further verified by melting curve analysis to confirm single peaks. The *RPS25-1* gene (GenBank accession number: GU797554.1) was used as the reference gene. Total RNA was reverse transcribed using HiScript^®^ III RT SuperMix (Vazyme R212) in a 20 µL reaction (50 °C for 15 min; 85 °C for 2 min). qRT-PCR was conducted with Hieff UNICON^®^ Universal Blue qPCR SYBR Green Master Mix (Yeasen Biotech Co., Ltd., Shanghai, China). Relative expression was calculated using the 2^⁻ΔΔCt^ method, with CK as the calibrator. For each gene, three independent biological replicates and three technical replicates were analyzed. Data are presented as mean ± SE (*n* = 3) of biological replicates.

## Results

### Antioxidant, osmotic, and photosynthetic responses to salt stress

Phenotypic observations revealed that salt stress induced visible damage to *G. jasminoides* leaves. After 15 days of treatment, leaves in the SS group exhibited distinct chlorosis and wilting compared to the green and healthy leaves in the CK group, indicating that the stress treatment was effective. Consistent with these phenotypic changes, the antioxidant defense system of *G. jasminoides* leaves exhibited pronounced and dynamic changes under salt stress. Activities of POD, SOD, and CAT, together with MDA accumulation, highlighted their central roles in oxidative stress regulation. POD activity increased steadily with stress intensity, with LS, MS, and SS treatments showing significant increases of 62.28%, 74.02%, and 197.51%, respectively, compared with CK. In contrast, SOD activity displayed a fluctuating pattern: LS and SS treatments showed significant increases of 38.04% and 39.26%, whereas MS treatment resulted in only an 18.40% increase. CAT activity decreased progressively, with reductions of 43.09%, 81.71%, and 83.85% in LS, MS, and SS, respectively. MDA accumulation partially mirrored SOD activity trends. Under SS treatment, MDA increased dramatically by 212.71%, indicating severe membrane lipid peroxidation, whereas LS showed a 57.65% increase and MS exhibited a slight 4.02% reduction **(**Fig. 1C**)**. These results suggest that POD and SOD are major contributors to antioxidant defense. However, the persistent decline in CAT activity likely impaired H_2_O_2_ detoxification, leading to elevated MDA accumulation under SS. The transient reduction in MDA at MS may indicate a temporary balance between ROS production and scavenging, although prolonged stress could disrupt this equilibrium [31, 32].

Chlorophyll fluorescence parameters also displayed distinct temporal patterns in response to salt stress. In the early stages (1–5 days), *F*_*v*_/*F*_*m*_ and *F*_*v*_/*F*_*o*_ showed no consistent significant differences compared to the control, indicating a relatively stable photosynthetic apparatus. However, at 15 days, salt stress significantly inhibited photosynthetic efficiency. Compared with CK, SS treatment caused a significant decrease in *F*_*v*_/*F*_*m*_ (4.30%) and *F*_*v*_/*F*_*o*_ (17.14%), accompanied by a significant increase in *F*_*o*_ of 12.86% (*P* < 0.05). This significant rise in *F*_*o*_ and decline in *F*_*v*_/*F*_*m*_ at Day 15 indicated the occurrence of photoinhibition and structural damage to PSII reaction centers, contrasting with the phenotype observed in early stages (Fig. S1) [48, 49].

Osmotic adjustment also exhibited differential responses. Soluble protein content decreased initially under LS but gradually recovered and peaked under SS. Free proline displayed a triphasic response, peaking significantly during MS before declining and rising again **(**Fig. 1B**)**. Together, these patterns suggest complementary roles in osmotic adjustment, with proline acting as an early stress indicator and soluble proteins contributing to sustained tolerance [50].

### Quality assessment of sequencing

**Fig. 2:**
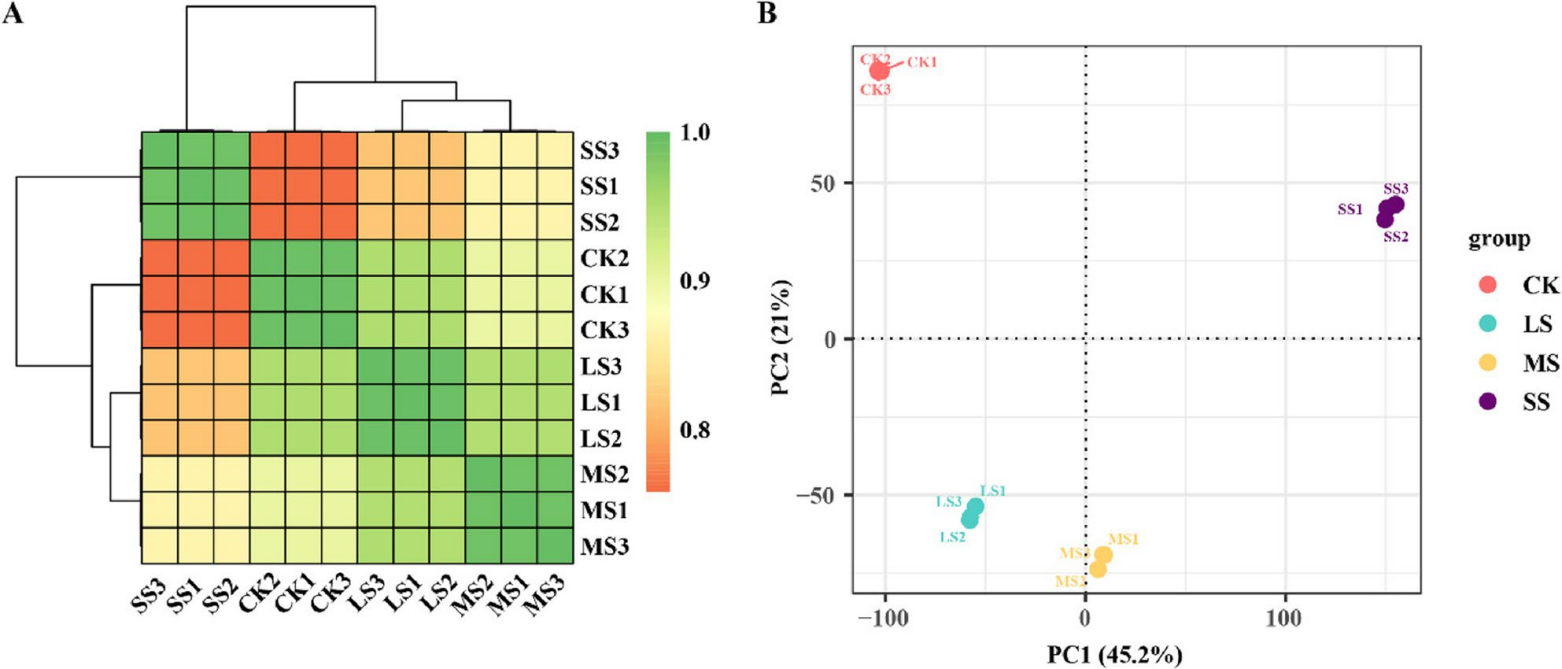
Transcriptome sequencing quality assessment. **A** Correlation studies among all treatments, including control group (CK), low salt (LS), medium salt (MS), severe salt (SS). **B** PCA

Transcriptome sequencing was conducted on 12 samples, including CK and the three salt stress treatments (LS, MS, SS). Sequencing quality was high, with Q20 values exceeding 95%, Q30 values above 92%, and genome mapping rates greater than 82.80% for all samples (Tab. S3). Pearson correlation coefficient (PCC) analysis indicated higher correlation within biological replicates than between treatments **(**Fig. 2A**)**. Principal component analysis (PCA) further confirmed the reliability of sample grouping, with PC1 and PC2 explaining 81% and 15% of total variance, respectively **(**Fig. 2B**)**. Across all samples, 25,951 expressed genes were detected, including 24,784 annotated genes and 1,167 novel predictions. A total of 30,383 new transcripts were identified, comprising 29,201 novel splice variants of known protein-coding genes and 1,182 transcripts corresponding to predicted new protein-coding genes. These statistics confirm that the sequencing data are of high quality and suitable for downstream analyses.

### Identification of DEGs

**Fig. 3:**
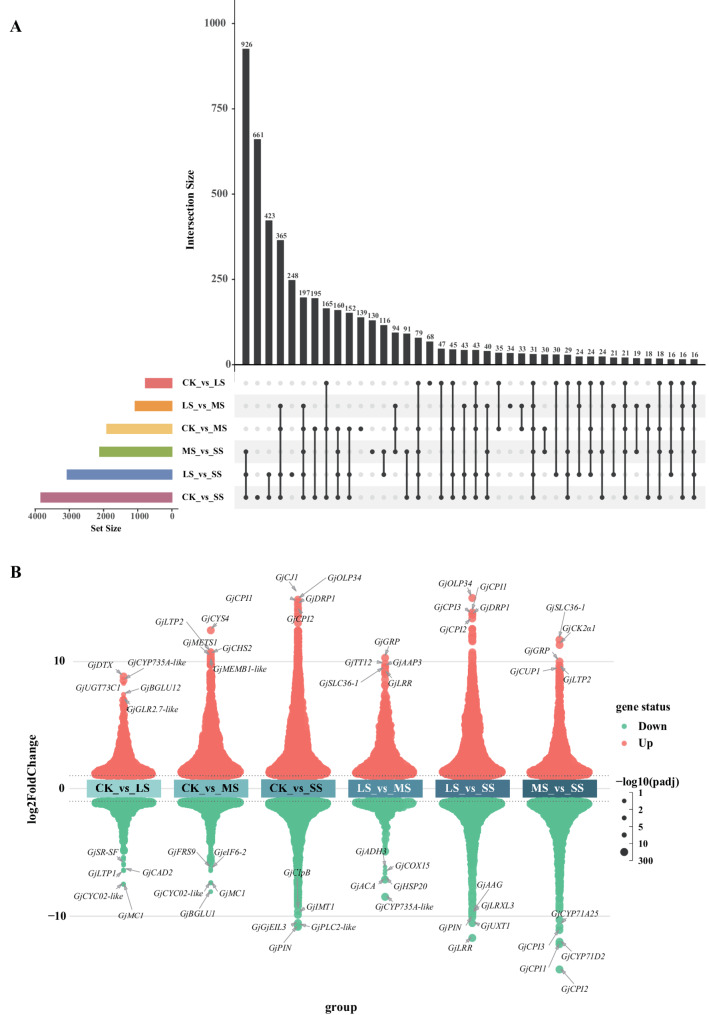
Differentially expressed genes (DEGs) in control, low salt, medium salt and severe salt. **A** Analysis of upset plot of DEGs in each treatment comparison. **B** The up-regulated and down-regulated DEGs and the top five significantly upregulated and downregulated genes

Six pairwise comparisons were conducted: CK vs. LS, CK vs. MS, CK vs. SS, LS vs. MS, LS vs. SS, and MS vs. SS. In total, 165 DEGs were shared across all comparisons. The most extensive transcriptional reprogramming was observed in CK vs. SS, which yielded 3,883 DEGs, with 2,452 up-regulated and 1,381 down-regulated genes. Substantial overlap was observed with CK vs. LS (549 shared genes) and CK vs. MS (1,510 shared genes) **(**Fig. 3A**)**. The number of DEGs increased with stress intensity, reflecting progressive transcriptome reprogramming. Specifically, CK vs. MS yielded 1,902 DEGs (1,614 up-regulated; 288 down-regulated), and CK vs. LS yielded 772 DEGs (557 up-regulated; 215 down-regulated). Additional comparisons identified 1,071 DEGs in LS vs. MS (992 up-regulated; 79 down-regulated), 3,063 in LS vs. SS (1,949 up-regulated; 1,113 down-regulated), and 2,111 in MS vs. SS (1,126 up-regulated; 985 down-regulated). The top five most significantly up- and down-regulated genes were identified **(**Fig. 3B, Tab. S4).

### Functional annotation of DEGs

GO functional enrichment analysis of DEGs from all six pairwise comparisons revealed that salt-responsive genes were primarily associated with stress response, hormone metabolism, ion transport, secondary metabolism, and cell wall biogenesis. Enriched categories included “response to bacterium,” “hormone metabolic process,” “monoatomic anion transport,” “secondary metabolic process,” and “plant-type secondary cell wall biogenesis” (Fig. S2).

**Fig. 4:**
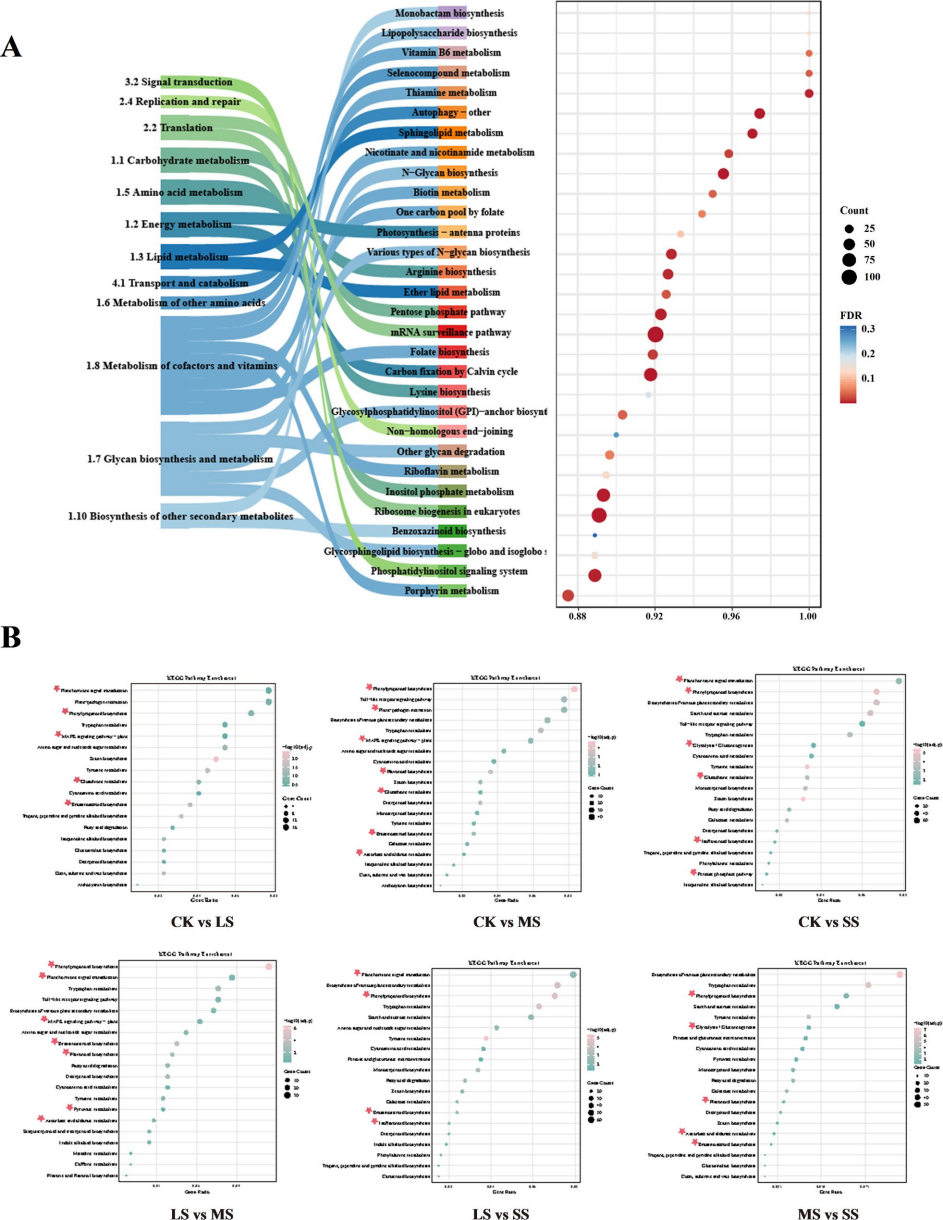
KEGG enrichment analysis of differentially expressed genes (DEGs). **A** KEGG enrichment analysis of all DEGs (res < 0.01). **B** KEGG enrichment analysis of differential genes between groups

KEGG annotation of DEGs (adjusted *P* < 0.01) showed enrichment in 12 major subclasses, including “Translation,” “Energy metabolism,” and “Transport and catabolism” **(**Fig. 4A**)**. KEGG enrichment analysis across the six comparisons identified “Phenylpropanoid biosynthesis” and “Tryptophan metabolism” as consistently enriched pathways (Fig. 4B; Tab. S5). The phenylpropanoid pathway produces flavonoid antioxidants that mitigate ROS accumulation, highlighting its importance in oxidative stress tolerance. Tryptophan metabolism, a precursor of indoleacetic acid (auxin), contributes to growth, development, and stress responses, and produces antioxidant metabolites that further enhance stress defense. Pathway-specific enrichment patterns were also observed. For example, “Glutathione metabolism” was significantly enriched in CK vs. LS and CK vs. SS but less so in other comparisons, suggesting stress-intensity-dependent regulation of this pathway.

### Candidate genes in salt stress

**Fig. 5:**
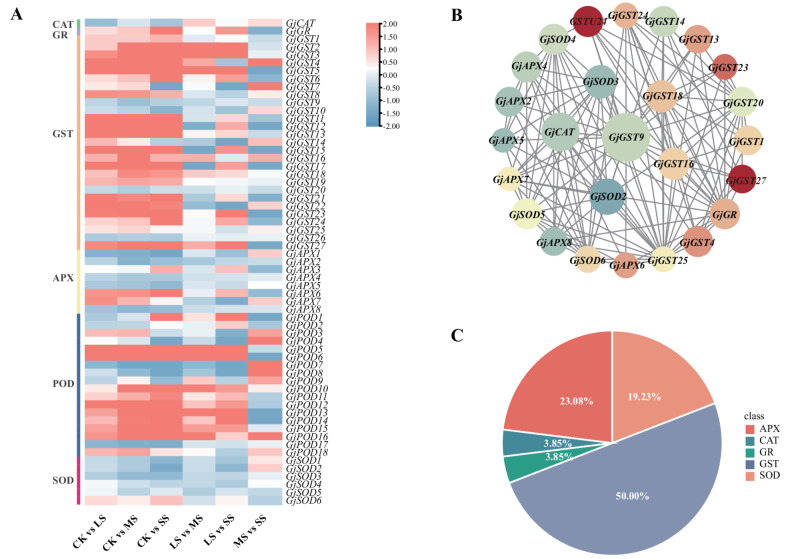
Protein-protein interaction (PPI) network of antioxidant-related genes in *G. jasminoides.* **A** Heatmap of up- and down-regulation of antioxidant enzyme genes. **B** PPI network of 26 antioxidant-related protein under salt stress. **C** Functional classification of 26 genes shown in panel (**B**)

Based on antioxidant and osmotic adjustment responses and functional enrichment analyses of DEGs, we identified 61 DEGs associated with antioxidant defense system, including isoforms of SOD, POD, CAT, glutathione reductase (GR), and glutathione S-transferase (GST) (Tab. S6). These families exhibited distinct expression trends. As salinity increased, *POD* genes were predominantly upregulated in a concentration-dependent manner. In particular, *GjPOD5*, *GjPOD6*, and *GjPOD13* showed progressively higher expression with increasing salt stress, peaking under SS, supporting a central role of POD-mediated H₂O₂ detoxification. SOD isoforms displayed divergent responses: *GjSOD6* increased steadily with salt concentration, whereas *GjSOD2* and *GjSOD3* declined, suggesting functional specialization among paralogs. The *GjCAT* gene was consistently suppressed, with expression further reduced at higher salinity, indicating diminished CAT-mediated ROS detoxification. Ascorbate peroxidase (APX) genes also showed contrasting responses: *GjAPX2*,* GjAPX5*, and *GjAPX8* were downregulated, whereas *GjAPX6* was markedly induced (log_2_FC = 2.69 under SS). GSTs represented the largest and most responsive group, with several strongly upregulated members. For example, *GjGST5* (log_2_FC = 13.89) and *GjGST27* (log_2_FC = 5.57) highlight their importance in glutathione-dependent ROS detoxification **(**Fig. 5A**)**. These patterns align with measured enzymatic activities and suggest coordinated regulation of antioxidant defenses under salt stress. The results indicate that *G. jasminoides* preferentially activates POD-mediated antioxidant pathways at higher stress intensities.

To further explore the regulatory mechanisms of antioxidant-related genes, we subjected the 61 DEGs to protein–protein interaction (PPI) and co-expression analyses **(**Fig. 5B**)**. The PPI network was constructed using *Arabidopsis thaliana* orthologs as references in the STRING database, yielding 26 genes with potential interactions. GSTs were the most abundant group (50.00%), followed by SODs (23.08%), APXs (19.23%), and CAT and GR (3.85% each), highlighting the importance of GSTs in salt stress responses **(**Fig. 5C**)**. Several highly connected genes were significantly upregulated, such as *GjGST4* (*Gj11A415T159*, log_2_FC = 3.09), *GjGST23* (*Gj9A71T113*, log_2_FC = 4.61), and *GjGST27* (*Gj9X778T41*, log_2_FC = 5.57), suggesting strong responsiveness and central regulatory roles (Tab. S7). In contrast, some highly connected but downregulated genes, such as *GjSOD2* and *GjAPX8*, may contribute to negative feedback regulation of ROS homeostasis.

### Calcium signaling and ROS production are central regulators of salt stress response

**Fig. 6:**
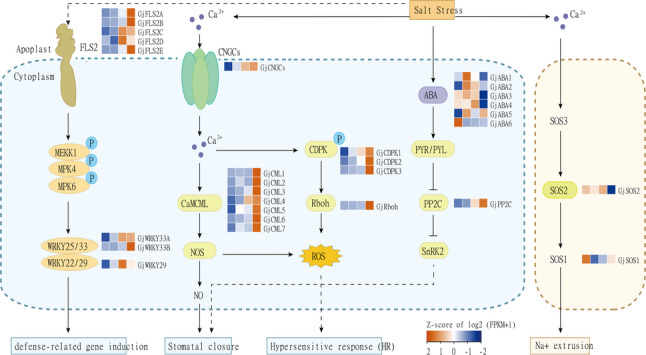
Schematic diagram of salt stress signaling pathway in the leaves of *G. jasminoides*, with overlaid gene expression profiles. Heatmaps represent the Z-score of log₂(FPKM + 1) expression values across four treatments: CK, LS, MS, SS. The color scale bar indicates the Z-score range from − 2 (blue, low expression) to + 2 (red, high expression)

Drawing on relevant literature and the expression patterns of DEGs detected in this study, we constructed a network diagram of the calcium signaling pathway and its associated gene expression changes in *G. jasminoides* leaves under salt stress **(**Fig. 6**)**. In total, 29 DEGs were identified, including genes associated with calcium signaling, ROS production, mitogen-activated protein kinase (MAPK) cascades, phytohormone signaling, and ionic homeostasis. These comprised six abscisic acid (ABA) biosynthesis and signaling genes (*GjABA1–6*), ten calcium-dependent protein kinase and calmodulin-like protein genes (*GjCDPK1–3*,* GjCML1–7*), one cyclic nucleotide-gated channels (*GjCNGCs*), five flavonol synthase genes (*GjFLS2A–E*), one protein phosphatase 2 C (*GjPP2C*), one respiratory burst oxidase homolog (*GjRboh*), two salt overly sensitive (SOS) pathway genes (*GjSOS1* and *GjSOS2*), and three WRKY transcription factors (*GjWRKY29*,* GjWRKY33A*, and *GjWRKY33B*) (Tab. S7). Salt stress is perceived as excessive sodium ion (Na^+^) accumulation, which rapidly elevates cytosolic calcium ion (Ca²^+^) levels and activates downstream signaling cascades [51]. Calcium signaling emerged as a central regulatory mechanism, supported by marked upregulation of *CNGC*, calmodulin/calmodulin-like (*CaM/CML*), calcium-dependent protein kinase (*CDPK*), and *Rboh* families [52]. *CNGCs* genes, encoding plasma membrane Ca²+-permeable channels, showed sustained upregulation, suggesting a key role in mediating Ca²^+^ influx and initiating intracellular signaling during salt stress. The concurrent upregulation of *CaM/CML*, *CDPK*, and *Rboh* indicates that elevated cytosolic Ca²^+^ activates ROS and nitric oxide (NO) production, driving cell wall remodeling and stomatal closure **(**Fig. 6; Tab. S8). The induction of *GjCNGCs* together with *GjRboh* suggests the existence of a “Ca²^+^ influx–*Rboh* activation–ROS burst” signaling cascade, consistent with findings in *Arabidopsis* and other plant species [53].

In parallel, flagellin-sensitive 2 (FLS2) receptor activates MAPK cascades [54]. These pathways mediate defense responses through modules such as *MEKK1*–*MPK4/MPK6*–*WRKY25/33* and *WRKY22/29*. In our study, two *WRKY25/33*-related genes and one *WRKY22/29* gene showed differential expression, suggesting the activation of distinct MAPK sub-pathways during salt stress (Tab. S7; Fig. S3).

Phytohormone signaling also contributes to salt adaptation [55]. ABA signaling plays a critical role in osmotic adjustment, ion balance, and ROS regulation [56, 57]. Salt-induced ABA accumulation is perceived by PYR/PYL receptors, which suppress *PP2C*, thereby activating sucrose non-fermenting 1-related protein kinase 2 (*SnRK2*). This cascade phosphorylates ion channels and induces stomatal closure. Interestingly, the specific upregulation of *GjPP2C* under salt stress may dampen *SnRK2* activity, potentially delaying stomatal closure to balance water loss with photosynthetic activity. For ion homeostasis, we analyzed genes in the SOS pathway. Although *GjSOS1* and *GjSOS2* did not show strong induction, their stable expression under high salinity suggests regulation at the post-translational level [58, 59]. Beyond the SOS pathway, vacuolar H^+^-ATPase (*V-ATPase*) genes were significantly upregulated, implying that vacuolar Na^+^ sequestration is a crucial tolerance mechanism in *G. jasminoides*. Additionally, the high expression of *annexin* (*ANN*) genes under low to moderate stress (MS) suggests that annexins may mediate Ca²^+^signaling and coordinate ion transport [60] (Tab. S9).

Collectively, these results support a comprehensive model in which Ca²^+^-mediated signaling integrates ROS production, MAPK activation, ABA signaling, and ion transport to orchestrate adaptive responses of *G. jasminoides* under salt stress **(**Fig. 6**)**.

### QRT-PCR validation of RNA-seq data

**Fig. 7:**
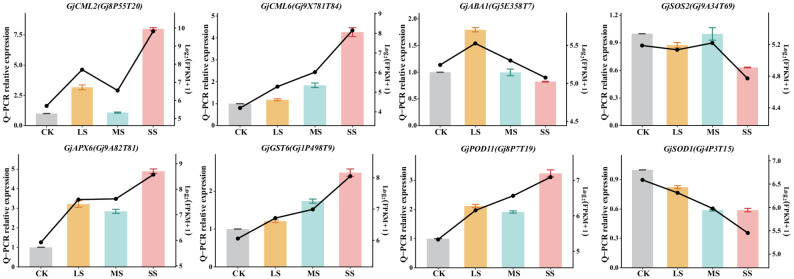
qRT-PCR validation of RNA-Seq data. Real-time PCR analysis of eight selected DEGs. These data are expressed as the mean ± SE (*n* = 3) relative to the control. The left Y-axis represents the relative expression level determined by qRT-PCR, and the right Y-axis represents the transcript abundance from RNA-seq, expressed as Log_2_(FPKM + 1) to allow for better visual comparison of trends

To validate RNA-seq results, eight DEGs were examined by qRT-PCR, covering three functional categories: antioxidant defense (*GjAPX6*,* GjGST6*,* GjPOD11*,* GjSOD1*), calcium signaling (*GjABA1*,* GjCML2*,* GjCML6*), and ion homeostasis (*GjSOS2*). Antioxidant enzyme-encoding genes *GjAPX6*,* GjGST6*, and *GjPOD11* were up-regulated in a concentration-dependent manner, whereas *GjSOD1* was down-regulated. Within the signaling category, *GjABA1* peaked under LS, whereas *GjCML2* and *GjCML6* were strongly induced under SS. *GjSOS2* expression was slightly reduced under SS relative to LS and MS. qRT-PCR expression patterns (bar charts) were compared with RNA-seq trends (line charts) across CK, LS, MS, and SS **(**Fig. 7**)**. Results were consistent between the two approaches, confirming the reliability of RNA-seq data in this study.

## Discussion

### Photosynthetic integrity and oxidative balance

Photosynthesis is a primary target of salt stress, and maintaining its functionality is paramount for survival [61, 62]. Extensive research on model plants and woody perennials has established a consensus regarding the physiological cascade triggered by salt injury, particularly concerning photosynthetic damage and antioxidant responses [63–66]. Miller et al. [65] discovered in *Oryza sativa* and *A. thaliana* that salt stress disrupts the chloroplast electron transport chain, precipitating ROS accumulation and activating nuclear-encoded antioxidant genes via retrograde signaling pathways. Similar patterns have been reported in ecologically or phylogenetically related woody crops. For instance, Ramalho et al. [67] investigated the stress response of coffee (*Coffea* spp.), a member of the Rubiaceae family, demonstrating that abiotic stress induces stomatal limitations and impairs C-assimilation, thereby triggering a strong antioxidative response to mitigate oxidative damage. Similarly, Ye et al. [68] observed that *Ginkgo biloba* counters salinity-induced PSII inhibition by mobilizing synergistic antioxidant mechanisms to preserve cellular integrity. Salt-tolerant varieties mitigated this damage by rapidly upregulating SOD and POD activities. Collectively, these findings underscore that the synergy between photosynthetic integrity and robust enzymatic defenses constitutes a cornerstone of salt tolerance [69].

To elucidate the specific adaptive strategies of *G. jasminoides*, we simultaneously monitored photosynthetic indicators and ROS-related markers temporally. Results indicated that during the early phase (Days 1–5), the photosynthetic apparatus remained relatively stable. However, a distinct physiological turning point occurred on Day 15, characterized by a precipitous decline in *F*_*v*_/*F*_*m*_ alongside a sharp rise in *F*_*o*_ (Fig. S1). This shift signifies a transition from short-term acclimation to significant physiological stress [70]. Based on these multi-time-point observations, we selected sampling on day 15 to capture the transcriptomic signature associated with this specific stress state, rather than the transient shock responses characteristic of earlier stages [71].

Consistent with the observed photosystem damage, transcriptomic analysis at Day 15 revealed the significant upregulation of specific *GjGST4/27* and *GjPOD6/18* genes. This transcriptional response strongly paralleled the marked increase in POD enzyme activity, demonstrating tight coordination between gene expression and enzymatic function **(**Fig. 1C**)**. However, the CAT pathway exhibited a divergent regulatory pattern: despite the activation of other antioxidants, CAT activity remained suppressed. This likely compromised cellular H₂O₂ scavenging capacity, contributing to the significant accumulation of MDA [72]. In summary, salt adaptation in *G. jasminoides* involves a trade-off between POD/GST activation and CAT suppression, which directly influences the extent of oxidative damage.

### Divergence in osmolyte accumulation and ionic homeostasis

In addition to causing oxidative damage, salt stress significantly reduces extracellular water potential, forcing plants to accumulate osmotic regulators to maintain cell turgor and water uptake [72]. *G. jasminoides* displayed distinct strategies under varying salinities. Free proline followed a triphasic pattern that peaked at moderate stress before declining, whereas soluble proteins increased progressively to reach a maximum under severe stress. Transcriptomic analysis provides metabolic evidence for this strategic transition. KEGG enrichment results indicated that “glutathione metabolism” was significantly enriched under low-salt stress (CK vs. LS), consistent with early antioxidant defenses (e.g., *GST* genes). Conversely, under severe stress (CK vs. SS), metabolic fluxes significantly shifted toward “starch and sucrose metabolism” and the “biosynthesis of various plant secondary metabolites.” This suggests that under high-salinity conditions, *G. jasminoides* mobilizes soluble sugars, secondary compounds, and proteins for coordinated accumulation to preserve osmotic balance and integrity.

Compared to annual crops, the osmotic regulation strategy of *G. jasminoides* exhibits distinct characteristics. For instance, in wheat (*Triticum aestivum*) and barley (*Hordeum vulgare*), proline typically accumulates in a nearly linear fashion with increasing stress intensity to maintain turgor pressure throughout the entire growth cycle [73]. However, in *G. jasminoides*, the later decline in proline suggests it primarily functions as a transient signaling molecule or a short-term buffer. In contrast, the progressive accumulation of soluble proteins aligns with long-term adaptation patterns observed in woody plants such as *Populus euphratica* [74]. These perennial species often prioritize the synthesis of stable macromolecules in persistently saline environments to prolong leaf longevity and maintain cellular integrity [5].

Regarding ion homeostasis, Na⁺ efflux is typically mediated by the SOS signaling pathway via plasma membrane Na⁺/H⁺ antiporters [75]. Interestingly, our RNA-seq data revealed that despite the physiological requirement for ion transport, the transcriptional levels of *GjSOS1* and *GjSOS2* remained unchanged across treatments **(**Fig. 6**)**. This implies that *G. jasminoides* may rely more on the rapid activation of pre-existing transporters rather than *de novo* transcriptional upregulation to increase protein abundance. This observation aligns with the classical salt stress signaling model, wherein calcium-responsive kinase complexescan directly regulate the activity of transporters like *SOS1* through phosphorylation without altering their mRNA levels [52]. Consequently, ion homeostasis in *G. jasminoides* is likely maintained through such post-translational modification mechanisms. The significant upregulation of *GjCDPKs* in our dataset provides molecular support for this hypothesis, suggesting that *G. jasminoides* maintains ion balance primarily via post-translational regulation, enabling rapid and reversible modulation of ion transport activity.

### Integrating Ca^2+^–MAPK–hormone signaling

To coordinate the physiological adaptation processes described above, *G. jasminoides* employs a hierarchical signaling network. Signal perception and transduction constitute the initial steps in activating stress tolerance mechanisms. Established models posit that cytosolic Ca²⁺ signatures act as primary sensors for Na⁺ toxicity, triggering phosphorylation cascades [59]. In this study, this classical pathway was clearly activated, evidenced by the significant upregulation of a suite of calcium sensor genes, particularly calmodulin-like proteins (*GjCMLs*) and calcium-dependent protein kinases (*GjCDPKs*) (Fig. 6). Crucially, calcium signaling appears coupled with MAPK cascade activation. KEGG enrichment analysis revealed significant enrichment of the “MAPK signaling pathway” under moderate salt stress (CK vs. MS), suggesting that Ca²⁺ signaling may amplify defense responses via the MAPK module [18].

Downstream of the kinase cascade, transcription factors (TFs) serve as central hubs regulating functional genes. In our study, *WRKY* family genes exhibited pronounced transcriptional shifts. Tikhomirova et al. [76] highlighted *WRKY* TFs play a pivotal role in regulating antioxidant systems and enhancing resilience in woody species. Given that the upregulation of *GjWRKY33A/B* coincides with the expression peaks of *GjGSTs* and *GjPODs*, we hypothesize that *WRKY33* mediates the link between upstream signaling and ROS scavenging.

Hormonal regulation, particularly via the ABA pathway, is pivotal for balancing survival and growth under stress [56, 57]. Interestingly, we observed that *GjPP2C*, a key negative regulator of ABA signaling, was significantly upregulated under severe stress (. Combined with the significant decline in *GjABA3/4* expression under severe stress, this suggests that *G. jasminoides* may employ a compensatory negative feedback mechanism by upregulating *GjPP2C*. This mechanism likely dampens excessive ABA signaling, thereby preventing severe growth arrest. Such a strategy would enable woody plants to maintain basal metabolic and photosynthetic capacities under prolonged salinity, standing in contrast to the rapid, robust stomatal closure mechanism mediated by the *CNGC5*/*6*/*9*/*12*–*OST1*/*SnRK2.6* axis in *Arabidopsis* [77].

### Breeding potential and integrated stress-responsive network

Based on our integrated physiological and transcriptomic analyses, we propose a mechanistic model for salt adaptation in *G. jasminoides***(**Fig. 6**)**. Upon salt stress perception, cytoplasmic Ca²⁺ sensors (*GjCaM/CML*, *GjCDPK*) and the MAPK cascade initiate signaling. These upstream signals converge on *GjWRKY*, a pivotal integrator coordinating ROS mitigation via *GjPOD*/*GjGST* activation, and osmotic adjustment through the ABA–*GjPP2C* module and the proline-to-protein shift. The synergistic action of this multilayered signaling network enables *G. jasminoides* to maintain leaf vitality and metabolic integrity under prolonged ionic toxicity, exemplifying a steady-state adaptive mechanism characteristic of woody plants.

The ultimate goal of elucidating salt tolerance mechanisms is to identify elite genetic resources for molecular breeding. This study highlights several candidate genes with high potential for enhancing salt resilience in woody ornamental plants. Yu et al. [78] demonstrated that the overexpression of a WRKY transcription factor (*PeWRKY31*) from poplar (*Populus* × *euramericana*) significantly reduced ROS accumulation and improved membrane integrity in transgenic plants under salt stress. Regarding antioxidant genes, Roxas et al. [79] reported that overexpressing specific *GST* genes in tobacco enhanced ROS scavenging capacity and significantly improved salt tolerance. Similarly, Ji et al. [80] demonstrated that the heterologous expression of a glutathione S-transferase gene (*GsGST*) from wild soybean (*Glycine soja*) effectively enhanced salt tolerance and mitigated oxidative damage in transgenic plants. These cases provide cross-species corroboration for the application potential of the *GjGSTs* identified in this study. Therefore, developing molecular markers linked to genes associated with long-term salt tolerance in *G. jasminoides* will lay the foundation for implementing molecular marker-assisted selection (MAS) in woody plant breeding. However, further validation is required to definitively establish the regulatory roles of these candidate genes. Future validation via overexpression, RNAi, or *CRISPR*/*Cas9* in *G. jasminoides* or model plants is essential to confirm their regulatory roles and breeding value.

## Conclusion

This study integrated physiological and transcriptomic analyses to elucidate the responses of *G. jasminoides* leaves to four levels of salt stress (CK, LS, MS, SS). Antioxidant enzymes and osmolytes exhibited distinct temporal patterns, with POD and *GST* activation forming the core of ROS detoxification, whereas soluble proteins and proline provided complementary roles in osmotic adjustment and structural stabilization. Transcriptome profiling identified key regulators of Ca²^+^ signaling (*CNGCs*, *CaM*/*CMLs*, *CDPKs*), MAPK–WRKY cascades, and ABA signaling (PP2C–SnRK2), which together form a coordinated regulatory network for salt adaptation. The reliability of RNA-seq data was confirmed by qRT-PCR validation of eight representative genes spanning antioxidant defense, calcium signaling, and ion homeostasis. We propose a Ca²^+^–ROS–MAPK–ABA model in which converging signals fine-tune ROS homeostasis, ion balance, and stomatal regulation in a stress-intensity-dependent manner. Within this framework, WRKY transcription factors and *GST* family members emerged as promising salt-responsive candidates for functional validation and molecular breeding. Collectively, these findings establish *G. jasminoides* as a model for understanding salt adaptation in woody perennials and provide valuable genetic resources for developing salt-tolerant ornamental and crop plants.

## Supplementary Information

Supplementary Material 1.

## Data Availability

All the raw transcriptome sequences generated in this study have been deposited in the Genome Sequence Archive (GSA) at the National Genomics Data Center (NGDC), Beijing Institute of Genomics, Chinese Academy of Sciences, under BioProject accession number PRJCA042161 and GSA accession number CRA027779 (https://ngdc.cncb.ac.cn/gsa/browse/CRA027779).

## Abbreviations

RNA-seq: RNA sequencing
Fo: Minimal fluorescence
Fv: Variable fluorescence
Fm: Maximal fluorescence
PSⅡ: Photosystem II
DEGs: Differentially Expressed Genes
MAPK: Mitogen-Activated Protein Kinase
POD: Peroxidase
SOD: Superoxide Dismutase
CAT: Catalase
MDA: Malondialdehyde
CaM/CML: Calmodulin/Calmodulin-Like
CDPK: Calcium-Dependent Protein Kinase
GST: Glutathione S-transferase
APX: Ascorbate Peroxidase
GR: Glutathione Reductase
SOS: Salt Overly Sensitive
qRT-PCR: Quantitative Real-Time Polymerase Chain Reaction
ROS: Reactive Oxygen Species
ABA: Abscisic Acid
GO: Gene Ontology
KEGG: Kyoto Encyclopedia of Genes and Genomes
PP2C: Protein Phosphatase 2 C
SnRK2: Snf1-Related Protein Kinase 2
FPKM: Fragments Per Kilobase of transcript per Million mapped reads
