# Supplementary material for: Physiological and transcriptomic insights into the molecular mechanisms of salt stress adaptation in Gardenia jasminoides
Source: BMC Plant Biol. 2026 Jan 2;26:203. doi: 10.1186/s12870-025-08042-z (PMC12866529; doi:10.1186/s12870-025-08042-z)
Supplement: Supplementary file 1 — Supplementary Material 1. [file 12870_2025_8042_MOESM1_ESM.docx]

**Supplementary Data**

[**Figure S1. Chlorophyll fluorescence parameters of *G.jasminoides* under different salt stress treatment.** **The parameters measured include (A) minimal fluorescence (*F_o_*), (B) maximal fluorescence (*F_m_*), (C) maximum quantum yield of PSII (*F_v_*/*F_m_*), and (D) potential photochemical activity (*F_v_*/*F_o_*). Error bars represent the standard error (SE) of the mean from three biological replicates.** 2](#_Toc216290919)

[**Figure S2. GO enrichment analysis of differentially expressed genes (DEGs) across six salt stress comparison groups** 3](#_Toc216290920)

[**Figure S3. Enriched pathways related to salt stress: Plant-pathogen interaction and Plant hormone signal transduction** 4](#_Toc216290921)

[**Table S1. Experimental design of salt stress treatments** 5](#_Toc216290922)

[**Table S2. Primer sequences for qRT-PCR validation of target genes** 5](#_Toc216290923)

[**Table S3. Quality statistics of filtered RNA-seq reads** 6](#_Toc216290924)

[**Table S4. Top 10 significantly up/down-regulated DEGs in six salt stress comparison groups** 7](#_Toc216290925)

[**Table S5. Top 10 KEGG enrichment pathways in six salt stress comparison groups** 10](#_Toc216290926)

[**Table S6. Expression profiles of antioxidant enzyme-related genes under salt stress** 13](#_Toc216290927)

[**Table S7. Antioxidant-related DEGs used for protein-protein interaction (PPI) network construction** 16](#_Toc216290928)

[**Table S8. FPKM values of calcium signaling-related genes under different salt treatments (CK, LS, MS, SS)** 17](#_Toc216290929)

[**Table S9. Expression profiles of ion transport and homeostasis-related genes across salt stress comparison groups** 18](#_Toc216290930)

**
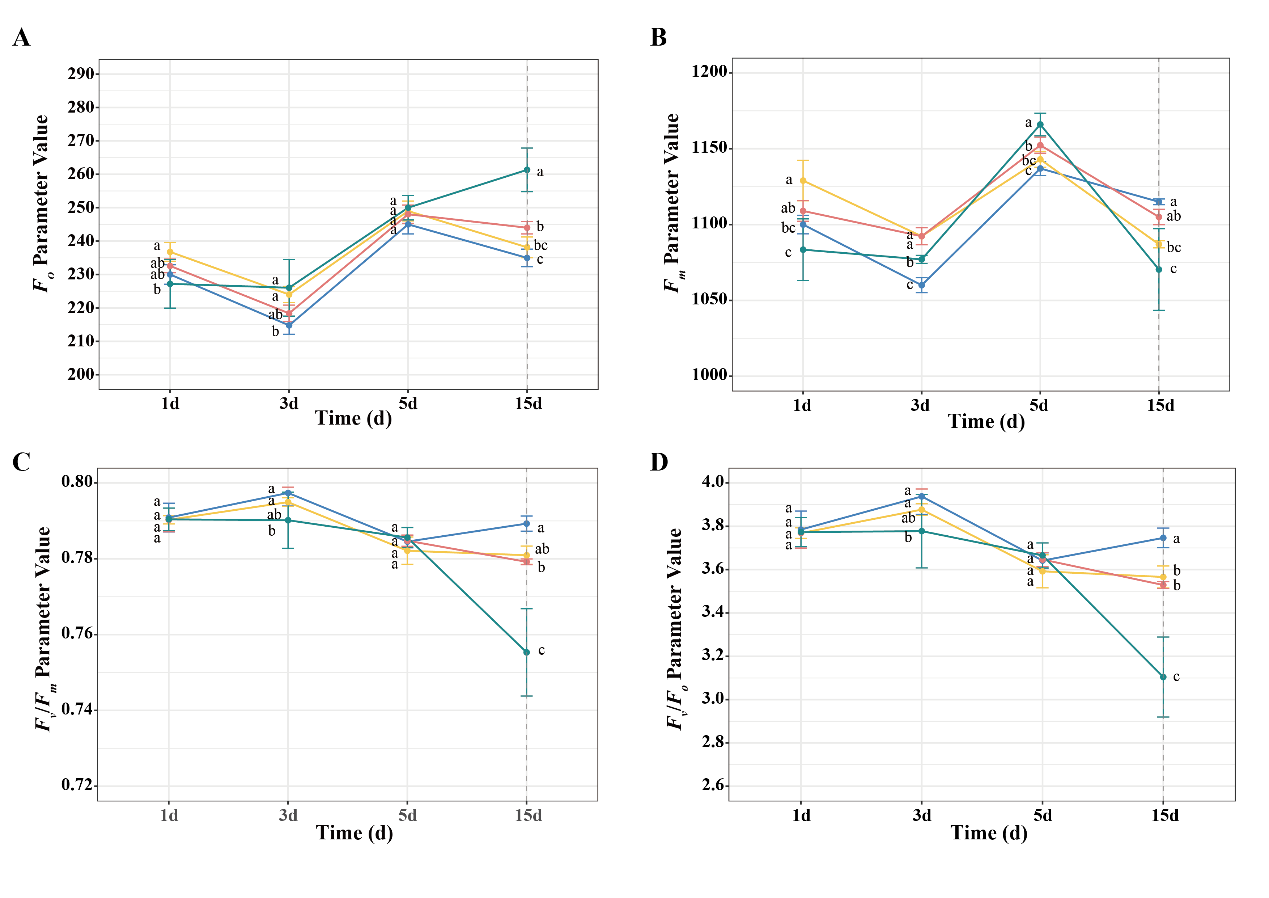
Figure S1. Chlorophyll fluorescence parameters of *G.jasminoides* under different salt stress treatment.** **The parameters measured include (A) minimal fluorescence (*F_o_*), (B) maximal fluorescence (*F_m_*), (C) maximum quantum yield of PSII (*F_v_*/*F_m_*), and (D) potential photochemical activity (*F_v_*/*F_o_*). Error bars represent the standard error (SE) of the mean from three biological replicates.**

**
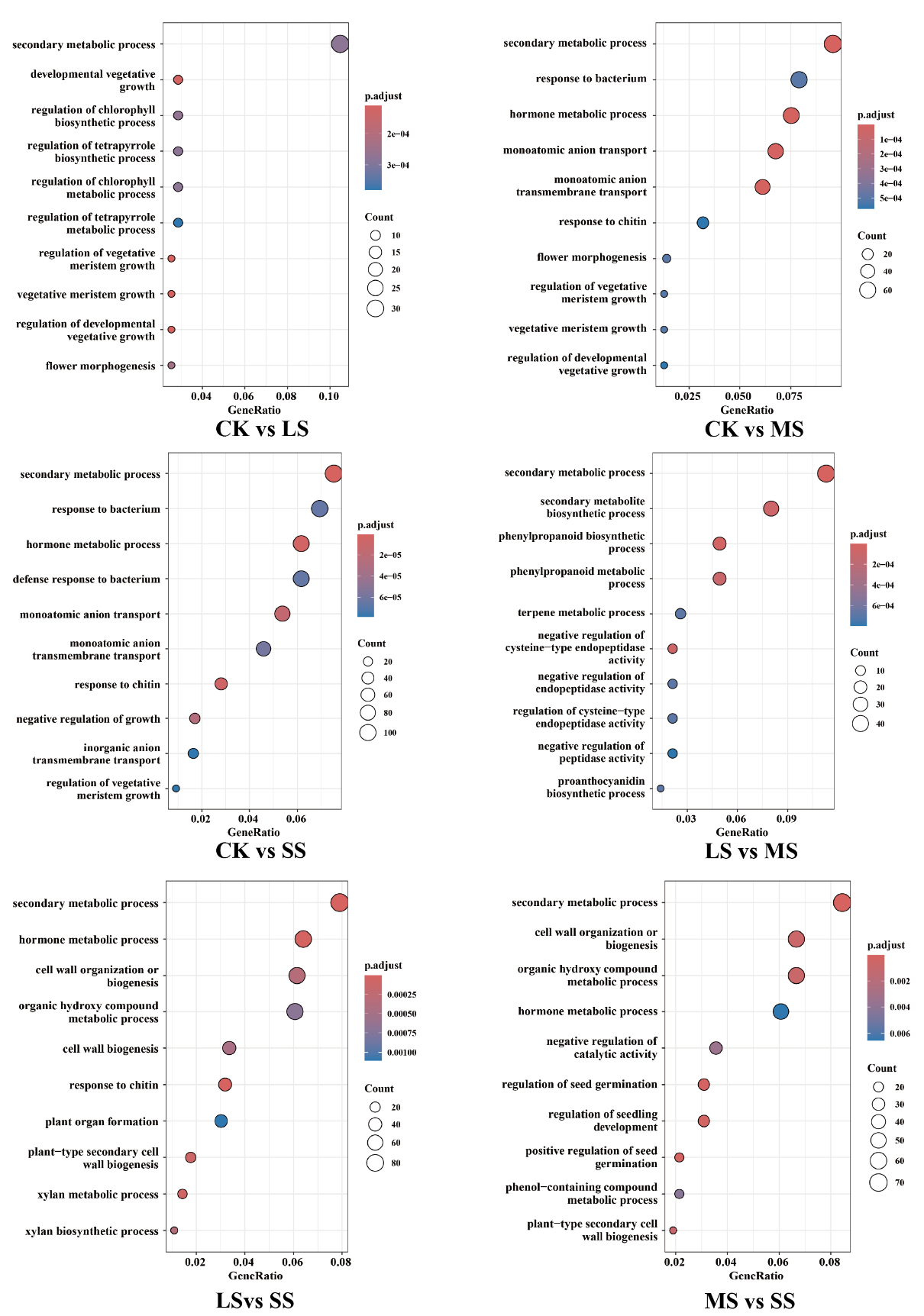
**

**Figure S2. GO enrichment analysis of differentially expressed genes (DEGs) across six salt stress comparison groups**

**
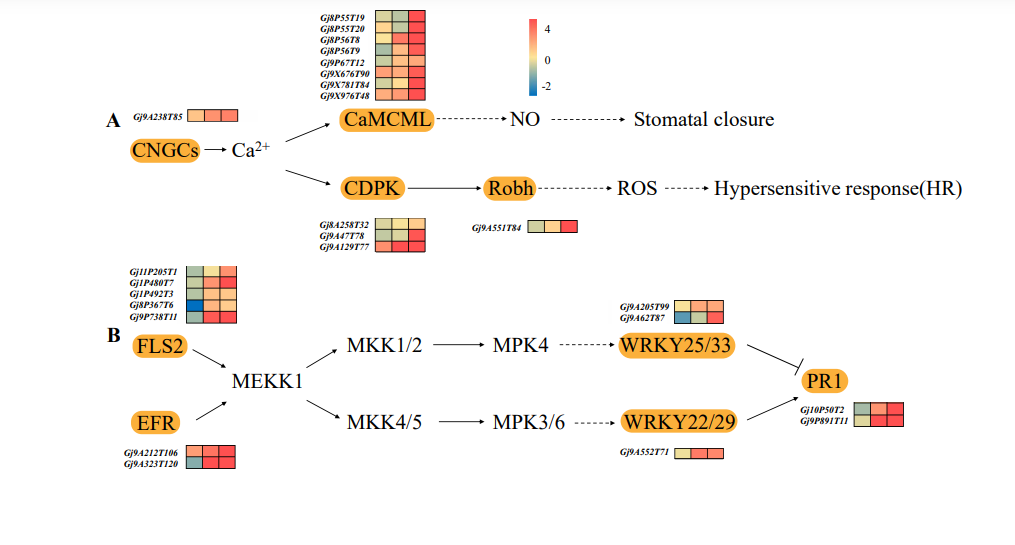

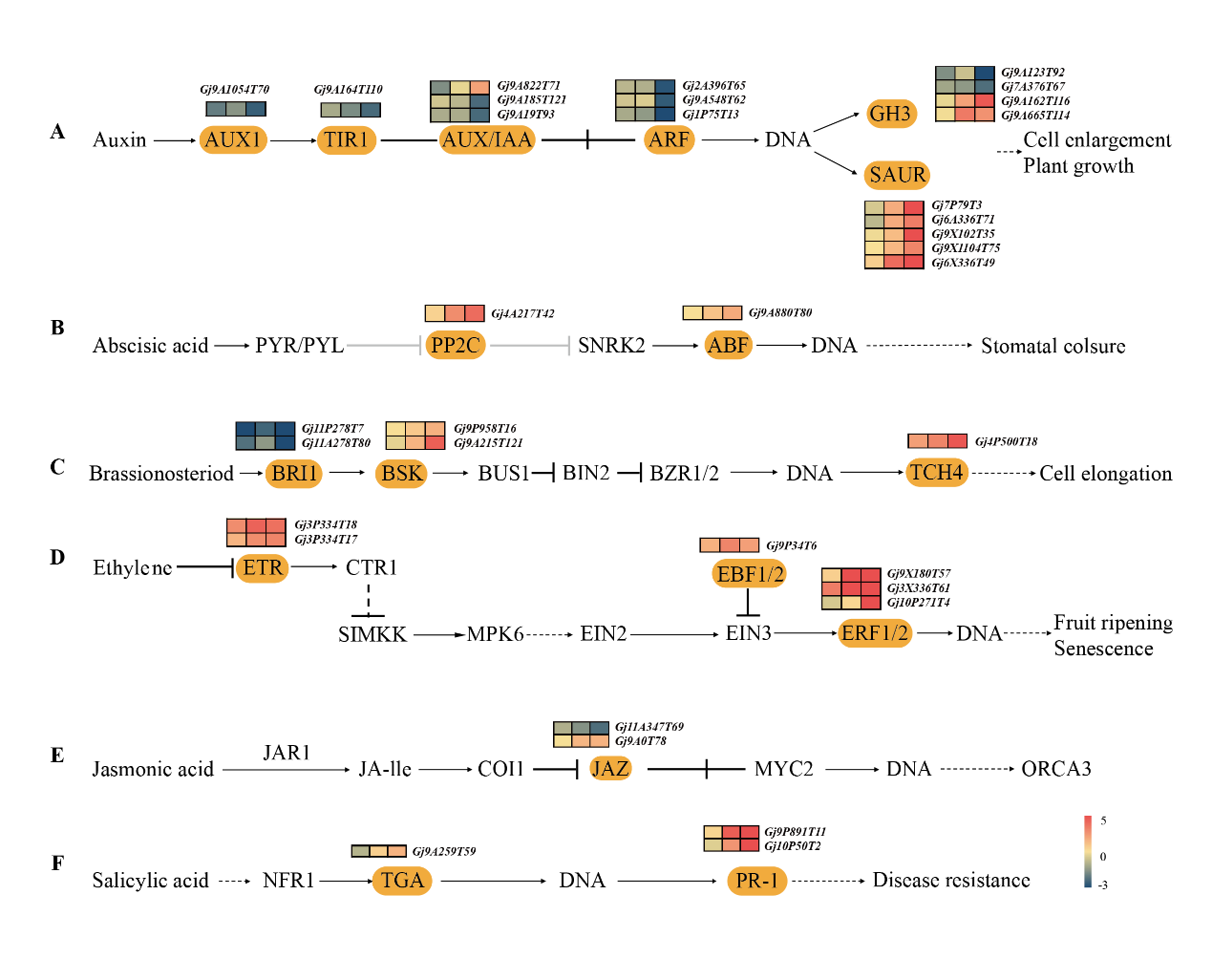
**

**Figure S3. Enriched pathways related to salt stress: Plant-pathogen interaction and Plant hormone signal transduction**

**Table S1. Experimental design of salt stress treatments**

| **Treatment Code** | **Treatment Description** | **NaCl Concentration (mmol·L⁻¹)** |
| --- | --- | --- |
| CK | Control (Deionized water) | 0 |
| LS | Low salt stress | 50 |
| MS | Moderate salt stress | 100 |
| SS | Severe salt stress | 200 |

**Table S2. Primer sequences for qRT-PCR validation of target genes**

| **Gene Name** | **Gene ID** | **Primer Sequence (5')** | **Primer Sequence (3')** | **Product Size** |
| --- | --- | --- | --- | --- |
| GjCML2 | Gj8P55T20 | CGAAAGCATAGGTGTCAACGC | GAGGCTGTCTTCTTCCTTCTCC | 160 |
| GjCML6 | Gj9X781T84 | CCCAACCACAACGAACAATG | TTTCATAACTCCGGCGAGCT | 148 |
| GjABA1 | Gj5E358T7 | TGTCGACATCCAAGATGAGC | AAGTCAACAGCATGGCTGAC | 120 |
| GjSOS2 | Gj9A34T69 | GAGTGTGGCCATCAAAGTCT | CCTCATGAAGCCTGACTA | 125 |
| GjAPX6 | Gj9A82T81 | AAGCTCTCGGAACTTGGTGG | GTCGGAATTGGAGGAAGGCA | 102 |
| GjGST6 | Gj1P498T9 | GAAGGCAGAGGTGTGGACAA | GTGCCTTGGTTTCCTTCTGC | 110 |
| GjPOD11 | Gj8P7T19 | AAGGGTCGTCTCATGTGCTG | GTTGCATTTCTCGTGGCGAA | 129 |
| GjSOD1 | Gj4P3T15 | CGGTTGTTGTGCATGCTGAT | GACGGATGACTGAAGCCCAA | 119 |
| GU797554.1 | GU797554.1 | AGTGGAGCAAGGGAAAGC | GACGGTCGGAAAGAACAG | 129 |

**Table S3. Quality statistics of filtered RNA-seq reads**

| **sample** | **Sample Description** | **Raw Read (M)** | **Clean Read (M)** | **Clean Base (Gb)** | **Q20 (%)** | **Q30 (%)** | **Clean Read Ratio (%)** | **Genome Mapping (%)** | **Gene Mapping (%)** |
| --- | --- | --- | --- | --- | --- | --- | --- | --- | --- |
| CK1 | Control group 1 | 45.57 | 42.77 | 6.42 | 97.69 | 93.7 | 93.84 | 87.69 | 62.4 |
| CK2 | Control group 2 | 45.57 | 42.86 | 6.43 | 97.54 | 93.37 | 94.05 | 87.97 | 62.95 |
| CK3 | Control group 3 | 45.57 | 42.7 | 6.4 | 97.65 | 93.63 | 93.69 | 87.86 | 62.92 |
| LS1 | Low salt 1 | 45.57 | 42.51 | 6.38 | 97.66 | 93.69 | 93.27 | 87.87 | 64.18 |
| LS2 | Low salt 2 | 45.57 | 42.62 | 6.39 | 97.65 | 93.63 | 93.51 | 88.44 | 65.34 |
| LS3 | Low salt 3 | 45.57 | 43.01 | 6.45 | 97.34 | 92.82 | 94.37 | 88.15 | 65.13 |
| MS1 | Medium salt 1 | 45.57 | 42.39 | 6.36 | 97.61 | 93.53 | 93.02 | 85.85 | 61.67 |
| MS2 | Medium salt 2 | 45.57 | 42.83 | 6.42 | 97.64 | 93.62 | 93.98 | 86.36 | 62.16 |
| MS3 | Medium salt 3 | 45.57 | 42.77 | 6.42 | 97.56 | 93.43 | 93.86 | 86.03 | 61.55 |
| SS1 | Severe salt 1 | 45.57 | 42.68 | 6.4 | 97.59 | 93.48 | 93.65 | 83.85 | 57.27 |
| SS2 | Severe salt 2 | 45.57 | 43.1 | 6.46 | 97.59 | 93.48 | 94.56 | 84.21 | 57.86 |
| SS3 | Severe salt 3 | 45.44 | 42.31 | 6.35 | 97.6 | 92.16 | 93.11 | 82.8 | 56.65 |

**Table S4. Top 10 significantly up/down-regulated DEGs in six salt stress comparison groups**

| **group** | **Gene ID** | **blastp** | **Gene Name** | **baseMean** | **log2FC** | **lfcSE** | **stat** | **pvalue** | **padj** | **gene_status** |
| --- | --- | --- | --- | --- | --- | --- | --- | --- | --- | --- |
| CK vs LS | *Gj10A244T70* | ProteinDETOXIFICATION27 | GjDTX | 74.00318 | 8.7667 | 1.186708 | 7.387409 | 1.50E-13 | 8.26E-13 | Up |
|  | *Gj9A717T107* | cytokinintrans-hydroxylase | GjCYP72A15 | 16.12199 | 8.457211 | 1.222958 | 6.915373 | 4.67E-12 | 2.36E-11 | Up |
|  | *Gj9X606T56* | UDP-glucosyltransferase73C | GjUGT73C1 | 31.05696 | 8.236617 | 1.547396 | 5.322888 | 1.02E-07 | 3.76E-07 | Up |
|  | *Gj5P89T2* | Beta-glucosidase12 | GjBGLU12 | 54.23504 | 7.358898 | 1.19212 | 6.172951 | 6.70E-10 | 2.93E-09 | Up |
|  | *Gj4A496T81* | Glutamatereceptor2.7 | GjGLR3 | 58.68794 | 7.060123 | 1.197254 | 5.89693 | 3.70E-09 | 1.53E-08 | Up |
|  | *Gj9A900T115* | Metacaspase-1 | GjMC1 | 10.78588 | -7.50392 | 2.428856 | -3.08949 | 0.002005 | 0.004528 | Down |
|  | *Gj9A735T75* | CYC02protein-like | GjCYC02-like | 7.483528 | -7.48741 | 1.283537 | -5.83342 | 5.43E-09 | 2.22E-08 | Down |
|  | *Gj3X293T78* | LipidTransferProtein | GjLTP1 | 4.652396 | -6.42154 | 1.342749 | -4.78238 | 1.73E-06 | 5.68E-06 | Down |
|  | *Gj10A265T43* | Cinnamylalcoholdehydrogenase2 | GjCAD2 | 6.981989 | -6.3868 | 1.954216 | -3.26822 | 0.001082 | 0.002546 | Down |
|  | *Gj6P0T7* | serine/arginine-richsplicingfactor | GjSR | 5.661897 | -5.99063 | 2.287028 | -2.61939 | 0.008809 | 0.017745 | Down |
| CK vs MS | *Gj3X279T22* | Cysteineproteinaseinhibitor4 | GjCYS-4 | 303.3174 | 12.38374 | 1.180542 | 10.48988 | 9.61E-26 | 6.17E-25 | Up |
|  | *Gj6X318T70* | Non-specificlipid-transferprotein1 | GjLTP1 | 149.0452 | 10.66841 | 1.181915 | 9.026378 | 1.77E-19 | 9.39E-19 | Up |
|  | *Gj9A758T75* | Chalconesynthase2; | GjCHS2 | 134.9735 | 10.52522 | 1.182337 | 8.902046 | 5.48E-19 | 2.84E-18 | Up |
|  | *Gj9A170T81* | METabolic Synthase1 | GjMETS1 | 424.9947 | 10.32569 | 1.204911 | 8.569675 | 1.04E-17 | 5.15E-17 | Up |
|  | *Gj9A180T127* | MEMbrane-associated Bindingprotein1 | GjMEMB1 | 421.6438 | 10.23803 | 0.83491 | 12.26244 | 1.44E-34 | 1.17E-33 | Up |
|  | *Gj2A113T45* | Oleuropeinbeta-glucosidase | GjBGLU | 36.4012 | -8.07923 | 2.883417 | -2.80196 | 0.0050793 | 0.009393 | Down |
|  | *Gj9A900T115* | Metacaspase-1 | GjMC2 | 10.78588 | -7.39145 | 2.428856 | -3.04318 | 0.0023409 | 0.004553 | Down |
|  | *Gj9A735T75* | CYC02protein-like | GjCYC02-like | 7.483528 | -7.37493 | 1.283537 | -5.74579 | 9.15E-09 | 2.92E-08 | Down |
|  | *Gj10A280T87* | eukaryotictranslationinitiationfactor6 | GjeIF6-2 | 8.921347 | -6.43584 | 2.18831 | -2.94101 | 0.0032714 | 0.006222 | Down |
|  | *Gj11A100T75* | FAR1-RELATEDSEQUENCE | GjFRS9 | 3.817268 | -6.37455 | 1.407164 | -4.53007 | 5.90E-06 | 1.52E-05 | Down |
| CK vs SS | *Gj9A1019T81* | Endochitinase/AllergenPersa1 | GjCJI | 3587.744 | 15.27989 | 1.179079 | 12.95918 | 2.08E-38 | 1.08E-37 | Up |
|  | *Gj11P324T10* | Osmotin-LikeProtein | GjOLP34 | 11680.03 | 14.77748 | 0.652955 | 22.63171 | 2.11E-113 | 2.60E-112 | Up |
|  | *Gju12567A0T42* | Cysteine Protease Inhibitor1 | GjCPI1 | 1145.196 | 14.60764 | 1.178697 | 12.39304 | 2.85E-35 | 1.39E-34 | Up |
|  | *Gj3A205T26* | Desiccation-Related Protein1 | GjDRP1 | 988.5375 | 14.39315 | 1.178707 | 12.21097 | 2.72E-34 | 1.30E-33 | Up |
|  | *Gj9A740T91* | Cysteine Protease Inhibitor1 | GjCPI2 | 829.5135 | 14.14239 | 1.181071 | 11.9742 | 4.85E-33 | 2.27E-32 | Up |
|  | *Gj1A509T67* | Auxineffluxcarriercomponent5 | GjPIN | 347.3416 | -10.8322 | 1.179827 | -9.18121 | 4.26E-20 | 1.50E-19 | Down |
|  | *Gj9A1055T89* | phosphoinositidephospholipaseC2-like | GjPLC2 | 151.7361 | -10.726 | 1.184674 | -9.05397 | 1.38E-19 | 4.77E-19 | Down |
|  | *Gj11A389T29* | Ethylene-Insensitive3-Like3* | GjEIL3 | 174.3544 | -10.5348 | 1.181233 | -8.91847 | 4.73E-19 | 1.61E-18 | Down |
|  | *Gj11A234T86* | Indole-3-acetateMethyltransferase1 | GjIMT1 | 60.70255 | -9.88911 | 1.188307 | -8.32201 | 8.65E-17 | 2.75E-16 | Down |
|  | *Gj9A1056T73* | ClpproteaseATP-bindingsubunitB1 | GjClpB | 604.354 | -9.62829 | 0.647112 | -14.8789 | 4.52E-50 | 2.83E-49 | Down |
| LS vs MS | *Gj9A634T38* | Glycine-RichProtein1.8 | GjGRP | 49.99118 | 10.22746 | 1.191768 | 8.581749 | 9.34E-18 | 9.12E-17 | Up |
|  | *Gj7A13T27* | TRANSPARENTTESTA12 | GjTT12 | 36.11795 | 9.751533 | 1.19492 | 8.160824 | 3.33E-16 | 3.01E-15 | Up |
|  | *Gj9A856T112* | AminoAcidPermease3 | GjAAP3 | 144.4232 | 9.542072 | 1.024736 | 9.311733 | 1.26E-20 | 1.42E-19 | Up |
|  | *Gj1P15T16* | SoluteCarrierFamily36Member1 | GjSLC36-1 | 163.5742 | 9.491697 | 0.837972 | 11.32698 | 9.65E-30 | 1.57E-28 | Up |
|  | *Gj9A41T69* | Leucine-RichRepeatExtensin-Like4 | GjLRR | 25.59052 | 9.236132 | 1.203658 | 7.673387 | 1.68E-14 | 1.36E-13 | Up |
|  | *Gj9A717T107* | cytokinintrans-hydroxylase | CYP735A | 16.12199 | -8.48192 | 1.222963 | -6.93554 | 4.05E-12 | 2.83E-11 | Down |
|  | *Gj2P27T7* | Auto-inhibitedCa²⁺-ATPase1 | GjACA | 256.0804 | -7.12818 | 0.727102 | -9.80355 | 1.09E-22 | 1.35E-21 | Down |
|  | *Gj11A433T109* | ChloroplasticsmallHeatShockProtein20 | HSP20 | 17.55508 | -7.02235 | 1.216547 | -5.77236 | 7.82E-09 | 4.25E-08 | Down |
|  | *Gj9P1029T26* | CytochromecOxidaseAssemblyProtein15 | GjCOX15 | 12.39768 | -6.6525 | 1.909815 | -3.48332 | 0.0004952 | 0.00148 | Down |
|  | *Gj9P312T6* | AlcoholDehydrogenaseClass-3 | ADH | 4.046686 | -6.41879 | 1.392896 | -4.60823 | 4.06E-06 | 1.64E-05 | Down |
| LS vs SS | *Gj11P324T10* | Osmotin-LikeProtein | GjOLP34 | 11680.03 | 14.9146 | 0.652931 | 22.84255 | 1.73E-115 | 2.95E-114 | Up |
|  | *Gju12567A0T42* | Cysteine Protease Inhibitor1 | GjCPI1 | 1145.196 | 13.78303 | 1.178702 | 11.6934 | 1.38E-31 | 7.95E-31 | Up |
|  | *Gj9X740T47* | Cysteine Protease Inhibitor1 | GjCPI1 | 532.2126 | 13.63934 | 1.183191 | 11.52759 | 9.58E-31 | 5.41E-30 | Up |
|  | *Gj3A205T26* | Desiccation-Related Protein1 | GjDRP1 | 988.5375 | 13.56855 | 1.178712 | 11.51134 | 1.16E-30 | 6.52E-30 | Up |
|  | *Gj9A740T91* | Cysteine Protease Inhibitor1 | GjCPI2 | 829.5135 | 13.31778 | 1.181076 | 11.27597 | 1.72E-29 | 9.45E-29 | Up |
|  | *Gj6A286T38* | Leucine-RichRepeatExtensin-Like4 | GjLRR | 220.7269 | -11.7012 | 1.18098 | -9.90806 | 3.84E-23 | 1.79E-22 | Down |
|  | *Gj9A238T81* | UDP-XyloseTransporter1 | GjUXT1 | 133.2026 | -10.547 | 1.18224 | -8.92119 | 4.61E-19 | 1.91E-18 | Down |
|  | *Gj1A509T67* | Auxineffluxcarriercomponent5 | GjPIN | 347.3416 | -10.0714 | 1.180046 | -8.53477 | 1.40E-17 | 5.49E-17 | Down |
|  | *Gj8P36T7* | Leucine-RichrepeateXtensin-Like3 | GjLRXL3 | 75.27201 | -9.88681 | 1.186776 | -8.33082 | 8.03E-17 | 3.05E-16 | Down |
|  | *Gj11A395T73* | Alkyl-AdenineDNAGlycosylase | GjAAG | 65.37746 | -9.79604 | 1.187444 | -8.24969 | 1.59E-16 | 5.98E-16 | Down |
| MS vs SS | *Gj1P15T16* | SoluteCarrierFamily36Member1 | GjSLC36-1 | 163.5742 | 11.64323 | 1.181505 | 9.854574 | 6.55E-23 | 3.78E-22 | Up |
|  | *Gj9A349T72* | CaseinKinase2alpha1 | GjCK2α1 | 127.2147 | 11.24139 | 1.182447 | 9.506883 | 1.96E-21 | 1.08E-20 | Up |
|  | *Gj9A634T38* | Glycine-RichProtein1.8 | GjGRP | 49.99118 | 9.936441 | 1.191768 | 8.33756 | 7.58E-17 | 3.48E-16 | Up |
|  | *Gj6X318T70* | Non-specificlipid-transferprotein1 | GjLTP1 | 149.0452 | 9.618954 | 1.02474 | 9.38673 | 6.19E-21 | 3.33E-20 | Up |
|  | *Gj2A352T52* | Cupredoxin1 | GjCUP1 | 83.86152 | 9.410121 | 1.185579 | 7.937154 | 2.07E-15 | 8.99E-15 | Up |
|  | *Gj9A740T91* | Cysteine Protease Inhibitor1 | GjCPI2 | 829.5135 | -14.1671 | 1.181076 | -11.9951 | 3.77E-33 | 2.89E-32 | Down |
|  | *Gju12567A0T42* | Cysteine Protease Inhibitor1 | GjCPI1 | 1145.196 | -12.1897 | 0.833995 | -14.6161 | 2.22E-48 | 2.36E-47 | Down |
|  | *Gj3A83T32* | CytochromeP45071D2 | GjCYP71D2 | 184.8041 | -12.001 | 1.181227 | -10.1598 | 3.00E-24 | 1.81E-23 | Down |
|  | *Gj9X740T47* | Cysteine Protease Inhibitor1 | GjCPI1 | 532.2126 | -11.0842 | 0.84033 | -13.1903 | 9.98E-40 | 8.87E-39 | Down |
|  | *Gj3A84T30* | CytochromeP45071A25 | GjCYP71A25 | 158.0358 | -10.8132 | 1.182358 | -9.14542 | 5.94E-20 | 3.09E-19 | Down |

**Table S5. Top 10 KEGG enrichment pathways in six salt stress comparison groups**

| **Category** | **KEGG Pathway** | **KEGG Num** | **DEG** | **Gene Ratio** | **pvalue** | **qvalue** |
| --- | --- | --- | --- | --- | --- | --- |
| **CK vs LS** | Zeatin biosynthesis | ko00908 | 11 | 0.05 | 3.99E-05 | 0.003315 |
|  | Tyrosine metabolism | ko00350 | 10 | 0.045455 | 0.000522 | 0.021543 |
|  | Tropane, piperidine and pyridine alkaloid biosynthesis | ko00960 | 7 | 0.031818 | 0.000777 | 0.021543 |
|  | Brassinosteroid biosynthesis | ko00905 | 8 | 0.036364 | 0.001467 | 0.030499 |
|  | Cutin, suberine and wax biosynthesis | ko00073 | 5 | 0.022727 | 0.002203 | 0.03664 |
|  | Isoquinoline alkaloid biosynthesis | ko00950 | 5 | 0.022727 | 0.004656 | 0.064049 |
|  | Amino sugar and nucleotide sugar metabolism | ko00520 | 12 | 0.054545 | 0.005391 | 0.064049 |
|  | Glutathione metabolism | ko00480 | 9 | 0.040909 | 0.010067 | 0.10464 |
|  | Phenylpropanoid biosynthesis | ko00940 | 15 | 0.068182 | 0.014844 | 0.137153 |
|  | Glucosinolate biosynthesis | ko00966 | 5 | 0.022727 | 0.025667 | 0.213445 |
| **CK vs MS** | Phenylpropanoid biosynthesis | ko00940 | 44 | 0.083176 | 1.74E-07 | 1.69E-05 |
|  | Flavonoid biosynthesis | ko00941 | 19 | 0.035917 | 6.61E-06 | 0.00032 |
|  | Diterpenoid biosynthesis | ko00904 | 16 | 0.030246 | 2.08E-05 | 0.00067 |
|  | Tryptophan metabolism | ko00380 | 34 | 0.064272 | 3.8E-05 | 0.000919 |
|  | Toll-like receptor signaling pathway | ko04620 | 41 | 0.077505 | 0.000195 | 0.003775 |
|  | Zeatin biosynthesis | ko00908 | 16 | 0.030246 | 0.000301 | 0.004852 |
|  | Biosynthesis of various plant secondary metabolites | ko00999 | 36 | 0.068053 | 0.000512 | 0.007077 |
|  | Brassinosteroid biosynthesis | ko00905 | 14 | 0.026465 | 0.000628 | 0.007608 |
|  | Plant-pathogen interaction | ko04626 | 41 | 0.077505 | 0.001019 | 0.01096 |
|  | MAPK signaling pathway - plant | ko04016 | 31 | 0.058601 | 0.001826 | 0.017679 |
| **CK vs SS** | Zeatin biosynthesis | ko00908 | 32 | 0.031465 | 3.41E-08 | 3.17E-06 |
|  | Tyrosine metabolism | ko00350 | 34 | 0.033432 | 6.33E-08 | 3.17E-06 |
|  | Phenylpropanoid biosynthesis | ko00940 | 68 | 0.066863 | 2.9E-07 | 9.66E-06 |
|  | Biosynthesis of various plant secondary metabolites | ko00999 | 68 | 0.066863 | 1.9E-06 | 4.43E-05 |
|  | Starch and sucrose metabolism | ko00500 | 65 | 0.063913 | 2.22E-06 | 4.43E-05 |
|  | Monoterpenoid biosynthesis | ko00902 | 33 | 0.032448 | 1.1E-05 | 0.000183 |
|  | Tryptophan metabolism | ko00380 | 55 | 0.054081 | 2.04E-05 | 0.000285 |
|  | Galactose metabolism | ko00052 | 24 | 0.023599 | 2.28E-05 | 0.000285 |
|  | Glutathione metabolism | ko00480 | 34 | 0.033432 | 2.73E-05 | 0.000303 |
|  | Plant hormone signal transduction | ko04075 | 79 | 0.077679 | 0.000215 | 0.002155 |
| **LS vs MS** | Phenylpropanoid biosynthesis | ko00940 | 33 | 0.114187 | 4.17E-09 | 3.25E-07 |
|  | Brassinosteroid biosynthesis | ko00905 | 13 | 0.044983 | 4.52E-06 | 0.000176 |
|  | Diterpenoid biosynthesis | ko00904 | 11 | 0.038062 | 6.48E-05 | 0.001657 |
|  | Tryptophan metabolism | ko00380 | 22 | 0.076125 | 9.07E-05 | 0.001657 |
|  | Flavonoid biosynthesis | ko00941 | 12 | 0.041522 | 0.000106 | 0.001657 |
|  | Fatty acid degradation | ko00071 | 11 | 0.038062 | 0.000594 | 0.007713 |
|  | Amino sugar and nucleotide sugar metabolism | ko00520 | 15 | 0.051903 | 0.003108 | 0.034589 |
|  | Biosynthesis of various plant secondary metabolites | ko00999 | 21 | 0.072664 | 0.003617 | 0.034825 |
|  | Tyrosine metabolism | ko00350 | 10 | 0.034602 | 0.004024 | 0.034825 |
|  | Caffeine metabolism | ko00232 | 3 | 0.010381 | 0.006813 | 0.053069 |
| **LS vs SS** | Tyrosine metabolism | ko00350 | 30 | 0.037736 | 3.46E-08 | 3.53E-06 |
|  | Tryptophan metabolism | ko00380 | 50 | 0.062893 | 7.56E-07 | 2.81E-05 |
|  | Phenylpropanoid biosynthesis | ko00940 | 56 | 0.07044 | 8.25E-07 | 2.81E-05 |
|  | Biosynthesis of various plant secondary metabolites | ko00999 | 57 | 0.071698 | 1.84E-06 | 4.7E-05 |
|  | Monoterpenoid biosynthesis | ko00902 | 27 | 0.033962 | 3.96E-05 | 0.000809 |
|  | Fatty acid degradation | ko00071 | 22 | 0.027673 | 0.000116 | 0.00197 |
|  | Galactose metabolism | ko00052 | 19 | 0.023899 | 0.000179 | 0.002442 |
|  | Zeatin biosynthesis | ko00908 | 21 | 0.026415 | 0.000201 | 0.002442 |
|  | Brassinosteroid biosynthesis | ko00905 | 19 | 0.023899 | 0.000215 | 0.002442 |
|  | Amino sugar and nucleotide sugar metabolism | ko00520 | 34 | 0.042767 | 0.000342 | 0.003494 |
| **MS vs SS** | Biosynthesis of various plant secondary metabolites | ko00999 | 53 | 0.094474 | 4.6E-10 | 4.46E-08 |
|  | Tryptophan metabolism | ko00380 | 43 | 0.076649 | 2.22E-08 | 1.07E-06 |
|  | Tyrosine metabolism | ko00350 | 24 | 0.042781 | 1.17E-07 | 3.79E-06 |
|  | Galactose metabolism | ko00052 | 17 | 0.030303 | 2.26E-05 | 0.000489 |
|  | Fatty acid degradation | ko00071 | 19 | 0.033868 | 2.53E-05 | 0.000489 |
|  | Cutin, suberine and wax biosynthesis | ko00073 | 10 | 0.017825 | 7.47E-05 | 0.001205 |
|  | Diterpenoid biosynthesis | ko00904 | 15 | 0.026738 | 0.000161 | 0.002223 |
|  | Flavonoid biosynthesis | ko00941 | 16 | 0.02852 | 0.000578 | 0.006608 |
|  | Phenylpropanoid biosynthesis | ko00940 | 36 | 0.064171 | 0.000614 | 0.006608 |
|  | Monoterpenoid biosynthesis | ko00902 | 19 | 0.033868 | 0.000693 | 0.006711 |

**Table S6. Expression profiles of antioxidant enzyme-related genes under salt stress**

| **Abbreviation** | **Enzyme Number** | **Pathway Number** | **Gene ID** | **Gene Name** | **log2FoldChange** | | | | | | **Description** |
| --- | --- | --- | --- | --- | --- | --- | --- | --- | --- | --- | --- |
|  |  |  |  |  | **CK_vs_LS** | **CK_vs_MS** | **CK_vs_SS** | **LS_vs_MS** | **LS_vs_SS** | **MS_vs_SS** |  |
| CAT | 1.11.1.6 | K03781 | *Gj10A283T99* | *GjCAT* | -0.96 | -0.15 | -0.76 | 0.82 | 0.20 | 0.62 | catalase |
| GR | 1.8.1.7 | K00383 | *Gj9A755T53* | *GjGR* | 0.60 | 0.85 | 2.16 | 0.25 | 1.55 | -1.31 | glutathione reductase |
| GST | 2.5.1.18 | K00799 | *Gj10A276T42* | *GjGST1* | 0.83 | 0.81 | 1.12 | -0.02 | 0.29 | -0.31 | glutathione S-transferase |
|  |  |  | *Gj11A415T156* | *GjGST2* | 0.83 | 2.94 | 3.10 | 2.11 | 2.27 | -0.16 | glutathione S-transferase |
|  |  |  | *Gj11A415T158* | *GjGST3* | 1.68 | 3.78 | 3.73 | 2.10 | 2.05 | 0.05 | glutathione S-transferase |
|  |  |  | *Gj11A415T159* | *GjGST4* | 3.88 | 5.15 | 3.09 | 1.27 | -0.79 | 2.06 | glutathione S-transferase |
|  |  |  | *Gj1A131T68* | *GjGST5* | 4.08 | 8.93 | 13.89 | 4.85 | 9.81 | -4.96 | glutathione S-transferase |
|  |  |  | *Gj1P498T9* | *GjGST6* | 0.59 | 0.93 | 2.06 | 0.34 | 1.47 | -1.14 | glutathione S-transferase |
|  |  |  | *Gj3A338T76* | *GjGST7* | 0.45 | 0.64 | -2.47 | 0.19 | -2.92 | 3.11 | glutathione S-transferase |
|  |  |  | *Gj3P338T15* | *GjGST8* | 1.68 | 1.58 | 1.17 | -0.10 | -0.51 | 0.41 | glutathione S-transferase |
|  |  |  | *Gj6A113T35* | *GjGST9* | -0.48 | -0.91 | -0.57 | -0.43 | -0.10 | -0.34 | glutathione S-transferase |
|  |  |  | *Gj6A306T142* | *GjGST10* | -0.32 | -0.51 | -1.16 | -0.19 | -0.84 | 0.65 | glutathione S-transferase |
|  |  |  | *Gj6A39T148* | *GjGST11* | 3.45 | 3.30 | 4.10 | -0.15 | 0.66 | -0.81 | glutathione S-transferase |
|  |  |  | *Gj6P39T14* | *GjGST12* | 5.18 | 3.83 | 6.41 | -1.34 | 1.23 | -2.57 | glutathione S-transferase |
|  |  |  | *Gj6P39T17* | *GjGST13* | 1.98 | 2.31 | 2.65 | 0.33 | 0.67 | -0.34 | glutathione S-transferase |
|  |  |  | *Gj7A407T107* | *GjGST14* | 1.06 | 0.44 | -0.54 | -0.62 | -1.60 | 0.98 | glutathione S-transferase |
|  |  |  | *Gj7A407T112* | *GjGST15* | 5.18 | 3.42 | 6.82 | -1.76 | 1.64 | -3.40 | glutathione S-transferase |
|  |  |  | *Gj7A407T121* | *GjGST16* | 1.17 | 2.36 | 1.15 | 1.19 | -0.02 | 1.21 | glutathione S-transferase |
|  |  |  | *Gj7A410T66* | *GjGST17* | 6.90 | 5.07 | 8.34 | -1.84 | 1.44 | -3.27 | glutathione S-transferase |
|  |  |  | *Gj9A162T126* | *GjGST18* | 0.95 | 1.77 | 1.58 | 0.83 | 0.64 | 0.19 | glutathione S-transferase |
|  |  |  | *Gj9A560T114* | *GjGST19* | 1.12 | 1.34 | 1.25 | 0.22 | 0.14 | 0.08 | glutathione S-transferase |
|  |  |  | *Gj9A560T119* | *GjGST20* | -0.36 | -0.60 | -0.27 | -0.24 | 0.08 | -0.33 | glutathione S-transferase |
|  |  |  | *Gj9A71T111* | *GjGST21* | 2.48 | 1.82 | 3.12 | -0.66 | 0.64 | -1.29 | glutathione S-transferase |
|  |  |  | *Gj9A71T112* | *GjGST22* | 3.58 | 2.58 | 1.84 | -1.00 | -1.74 | 0.74 | glutathione S-transferase |
|  |  |  | *Gj9A71T113* | *GjGST23* | 1.94 | 2.23 | 4.61 | 0.29 | 2.67 | -2.38 | glutathione S-transferase |
|  |  |  | *Gj9A81T115* | *GjGST24* | 0.70 | 0.88 | 2.00 | 0.18 | 1.29 | -1.11 | glutathione S-transferase |
|  |  |  | *Gj9P560T11* | *GjGST25* | 0.53 | 0.70 | 0.32 | 0.17 | -0.21 | 0.38 | glutathione S-transferase |
|  |  |  | *Gj9P857T15* | *GjGST26* | -0.67 | -0.59 | -0.66 | 0.09 | 0.01 | 0.08 | glutathione S-transferase |
|  |  |  | *Gj9X778T41* | *GjGST27* | 1.78 | 2.98 | 5.57 | 1.21 | 3.79 | -2.59 | glutathione S-transferase |
| APX | 1.11.1.11 | K00434 | *Gj10A289T101* | *GjAPX1* | -1.21 | -1.31 | -2.11 | -0.10 | -0.90 | 0.80 | L-ascorbate peroxidase |
|  |  |  | *Gj8A387T77* | *GjAPX2* | -0.50 | -1.23 | -0.92 | -0.73 | -0.42 | -0.31 | L-ascorbate peroxidase |
|  |  |  | *Gj8A93T22* | *GjAPX3* | 0.26 | 0.14 | 1.19 | -0.12 | 0.94 | -1.05 | L-ascorbate peroxidase |
|  |  |  | *Gj8A94T33* | *GjAPX4* | -0.49 | -0.71 | -0.68 | -0.22 | -0.19 | -0.03 | L-ascorbate peroxidase |
|  |  |  | *Gj9A274T88* | *GjAPX5* | -0.70 | -0.80 | -1.05 | -0.10 | -0.34 | 0.24 | L-ascorbate peroxidase |
|  |  |  | *Gj9A82T81* | *GjAPX6* | 1.60 | 1.67 | 2.69 | 0.07 | 1.09 | -1.02 | L-ascorbate peroxidase |
|  |  |  | *Gj9P230T12* | *GjAPX7* | 1.69 | 1.12 | 0.33 | -0.57 | -1.37 | 0.80 | L-ascorbate peroxidase |
|  |  |  | *Gj9P861T15* | *GjAPX8* | -0.82 | -1.12 | -0.98 | -0.30 | -0.16 | -0.14 | L-ascorbate peroxidase |
| POD | 1.11.1.7 | K00430 | *Gj10A248T136* | *GjPOD1* | -0.66 | -0.14 | 2.33 | 0.52 | 2.99 | -2.47 | Peroxidase |
|  |  |  | *Gj10A287T108* | *GjPOD2* | -0.49 | -0.44 | 0.29 | 0.05 | 0.78 | -0.74 | Peroxidase |
|  |  |  | *Gj10A69T30* | *GjPOD3* | 1.02 | 1.11 | -0.26 | 0.08 | -1.28 | 1.37 | Peroxidase |
|  |  |  | *Gj1A23T101* | *GjPOD4* | 0.26 | -0.09 | -4.87 | -0.34 | -5.13 | 4.79 | Peroxidase |
|  |  |  | *Gj4A11T96* | *GjPOD5* | 4.15 | 6.36 | 6.69 | 2.21 | 2.54 | -0.33 | Peroxidase |
|  |  |  | *Gj4A503T94* | *GjPOD6* | 4.18 | 7.76 | 10.51 | 3.57 | 6.32 | -2.75 | Peroxidase |
|  |  |  | *Gj5A359T42* | *GjPOD7* | -1.13 | -2.23 | -6.68 | -1.11 | -5.55 | 4.44 | Peroxidase |
|  |  |  | *Gj5A360T47* | *GjPOD8* | -0.27 | -1.06 | -6.04 | -0.79 | -5.77 | 4.98 | Peroxidase |
|  |  |  | *Gj7A388T144* | *GjPOD9* | -0.48 | 0.35 | -1.01 | 0.83 | -0.53 | 1.36 | Peroxidase |
|  |  |  | *Gj8A7T97* | *GjPOD10* | 0.44 | 2.34 | 1.96 | 1.90 | 1.52 | 0.38 | Peroxidase |
|  |  |  | *Gj8P7T19* | *GjPOD11* | 0.74 | 1.20 | 1.80 | 0.46 | 1.06 | -0.59 | Peroxidase |
|  |  |  | *Gj9A1062T111* | *GjPOD12* | 2.01 | 2.91 | 3.55 | 0.89 | 1.54 | -0.64 | peroxidase |
|  |  |  | *Gj9A33T158* | *GjPOD13* | 1.41 | 3.44 | 5.74 | 2.03 | 4.33 | -2.30 | peroxidase |
|  |  |  | *Gj9A628T149* | *GjPOD14* | 1.07 | 1.95 | 3.99 | 0.88 | 2.92 | -2.05 | peroxidase |
|  |  |  | *Gj9A663T75* | *GjPOD15* | 1.40 | 2.88 | 2.94 | 1.47 | 1.53 | -0.06 | peroxidase |
|  |  |  | *Gj9A673T127* | *GjPOD16* | 1.62 | 4.45 | 2.38 | 2.84 | 0.76 | 2.08 | peroxidase |
|  |  |  | *Gj9A824T97* | *GjPOD17* | -1.14 | -1.29 | -1.34 | -0.15 | -0.21 | 0.05 | peroxidase |
|  |  |  | *Gj9X695T53* | *GjPOD18* | 1.10 | 1.39 | 0.51 | 0.29 | -0.59 | 0.88 | peroxidase |
| SOD2 | 1.15.1.1 | K04564 | *Gj4P3T15* | *GjSOD1* | -0.38 | -0.67 | -1.14 | -0.30 | -0.77 | 0.47 | superoxide dismutase, Cu-Zn family |
|  |  |  | *Gj4P3T21* | *GjSOD2* | -0.34 | -0.65 | -1.45 | -0.32 | -1.11 | 0.79 | superoxide dismutase, Cu-Zn family |
|  |  |  | *Gj8P333T14* | *GjSOD3* | -0.61 | -1.03 | -1.06 | -0.42 | -0.46 | 0.04 | superoxide dismutase, Fe-Mn family |
|  |  |  | *Gj9A1068T86* | *GjSOD4* | 0.17 | -0.24 | -0.47 | -0.41 | -0.64 | 0.23 | superoxide dismutase, Fe-Mn family |
|  |  |  | *Gj9A245T63* | *GjSOD5* | 0.12 | -0.49 | -0.07 | -0.62 | -0.19 | -0.42 | superoxide dismutase, Cu-Zn family |
|  |  |  | *Gj9P203T10* | *GjSOD6* | 0.61 | 0.40 | 0.94 | -0.21 | 0.33 | -0.54 | superoxide dismutase, Fe-Mn family |

**Table S7. Antioxidant-related DEGs used for protein-protein interaction (PPI) network construction**

| **Gene ID** | **Gene Name** | **log2FoldChange** | **padj** | **degree** |
| --- | --- | --- | --- | --- |
| *Gj10A276T42* | *GjGST1* | 1.117791 | 1.95E-35 | 5 |
| *Gj10A283T99* | *GjCAT* | -0.76465 | 1.75E-22 | 12 |
| *Gj11A415T159* | *GjGST4* | 3.092358 | 9.47E-16 | 6 |
| *Gj4P3T21* | *GjSOD2* | -1.44635 | 1.31E-18 | 10 |
| *Gj6A113T35* | *GjGST9* | -0.57081 | 1.67E-46 | 20 |
| *Gj6P39T17* | *GjGST13* | 2.651973 | 5.16E-89 | 4 |
| *Gj7A407T107* | *GjGST14* | -0.53703 | 4.76E-26 | 6 |
| *Gj7A407T121* | *GjGST16* | 1.146772 | 3.68E-46 | 7 |
| *Gj7A410T66* | *GjGST17* | 8.341649 | 3.78E-51 | 6 |
| *Gj8A387T77* | *GjAPX2* | -0.92133 | 4.77E-60 | 5 |
| *Gj8A94T33* | *GjAPX4* | -0.67623 | 1.65E-21 | 6 |
| *Gj8P333T14* | *GjSOD3* | -1.06312 | 1.44E-58 | 8 |
| *Gj9A1068T86* | *GjSOD4* | -0.47125 | 3.06E-62 | 6 |
| *Gj9A162T126* | *GjGST18* | 1.583124 | 1.27E-144 | 7 |
| *Gj9A245T63* | *GjSOD5* | -0.07053 | 0.031587 | 6 |
| *Gj9A274T88* | *GjAPX5* | -1.04509 | 1.12E-85 | 1 |
| *Gj9A560T119* | *GjGST20* | -0.27198 | 7.81E-08 | 5 |
| *Gj9A71T113* | *GjGST23* | 4.612366 | 2.63E-24 | 2 |
| *Gj9A755T53* | *GjGR* | 2.1593 | 0 | 6 |
| *Gj9A81T115* | *GjGST24* | 1.995785 | 2.85E-207 | 2 |
| *Gj9A82T81* | *GjAPX6* | 2.69484 | 0 | 2 |
| *Gj9P203T10* | *GjSOD6* | 0.938698 | 2.22E-158 | 3 |
| *Gj9P230T12* | *GjAPX7* | 0.325258 | 1.01E-32 | 1 |
| *Gj9P560T11* | *GjGST25* | 0.321154 | 0.03601 | 2 |
| *Gj9P861T15* | *GjAPX8* | -0.98217 | 7.45E-282 | 9 |
| *Gj9X778T41* | *GjGST27* | 5.569069 | 2.86E-64 | 7 |

**Table S8. FPKM values of calcium signaling-related genes under different salt treatments (CK, LS, MS, SS)**

| **Gena ID** | **Gene Name** | **CK** | **LS** | **MS** | **SS** |
| --- | --- | --- | --- | --- | --- |
| *Gj5E358T7* | *GjABA1* | 36.85 | 45.05333 | 38.41 | 32.71333333 |
| *Gj5E360T1* | *GjABA2* | 2.256667 | 4.06 | 3.586667 | 2.893333333 |
| *Gj8A12T60* | *GjABA3* | 838.5 | 948.7733 | 867.85 | 295.5266667 |
| *Gj8A12T62* | *GjABA4* | 314.2033 | 304.7367 | 395.1533 | 119.0266667 |
| *Gj9E813T2* | *GjABA5* | 19.47 | 22.41 | 20.9 | 22.11 |
| *Gj9P146T25* | *GjABA6* | 17.57 | 15.57 | 15.67 | 15.75333333 |
| *Gj8A258T32* | *GjCDPK1* | 1.726667 | 4.21 | 5.446667 | 7.236666667 |
| *Gj9A129T77* | *GjCDPK2* | 0.286667 | 2.736667 | 5.623333 | 14.23 |
| *Gj9A47T78* | *GjCDPK3* | 0.473333 | 0.92 | 1.14 | 7.126666667 |
| *Gj8P55T19* | *GjCML1* | 10.18333 | 27.79667 | 19.57333 | 478.79 |
| *Gj8P55T20* | *GjCML2* | 50.71667 | 206.2733 | 92.41333 | 900.9666667 |
| *Gj8P56T8* | *GjCML3* | 0.146667 | 1.14 | 2.86 | 10.97666667 |
| *Gj9P67T12* | *GjCML4* | 6.003333 | 12.08333 | 30.28667 | 36.79666667 |
| *Gj9X676T90* | *GjCML5* | 5.29 | 39.02333 | 33.53333 | 73.9 |
| *Gj9X781T84* | *GjCML6* | 17.29333 | 37.97667 | 63.92667 | 282.9366667 |
| *Gj9X976T48* | *GjCML7* | 0.053333 | 1.38 | 1.06 | 4.29 |
| *Gj9A238T85* | *GjCNGCs* | 1.456667 | 7.69 | 12.52667 | 13.97666667 |
| *Gj11P205T1* | *GjFLS2A* | 0.86 | 1.366667 | 2.613333 | 6.546666667 |
| *Gj1P480T7* | *GjFLS2B* | 0.083333 | 0.27 | 1.023333 | 6.273333333 |
| *Gj1P492T3* | *GjFLS2C* | 1.73 | 2.823333 | 8.63 | 7.536666667 |
| *Gj8P367T6* | *GjFLS2D* | 0.476667 | 0.01 | 1.823333 | 1.043333333 |
| *Gj9P738T11* | *GjFLS2E* | 0.046667 | 0.073333 | 1.133333 | 4.4 |
| *Gj4A217T42* | *GjPP2C* | 0.393333 | 1.006667 | 3.783333 | 6.443333333 |
| *Gj9A551T84* | *GjRboh* | 0.173333 | 0.393333 | 0.716667 | 11.63333333 |
| *Gj11A261T51* | *GjSOS1* | 21.84667 | 17.29 | 18.43 | 19.88 |
| *Gj9A34T69* | *GjSOS2* | 35.44 | 34.10333 | 36.25 | 26.32666667 |
| *Gj9A552T71* | *GjWRKY29* | 0.026667 | 0.283333 | 0.736667 | 0.413333333 |
| *Gj9A205T99* | *GjWRKY33A* | 15.36 | 51.01667 | 102.34 | 98.64666667 |
| *Gj9A62T87* | *GjWRKY33B* | 1.4 | 0.89 | 2.683333 | 18.41 |

**Table S9. Expression profiles of ion transport and homeostasis-related genes across salt stress comparison groups**

| **Gene ID** | **CK vs LS** | **CK vs MS** | **CK vs SS** | **LS vs MS** | **LS vs SS** | **MS vs SS** | **Abbreviation** |
| --- | --- | --- | --- | --- | --- | --- | --- |
| *Gj10A254T83* | 2.038441 | 2.114047 | 0.272103 | 0.075607 | -1.76634 | 1.84194443 | ANN |
| *Gj10A258T67* | -1.13548 | 0.297156 | -1.84841 | 1.432635 | -0.71293 | 2.1455615 | ANN |
| *Gj2A255T23* | 5.625983 | 5.963759 | 4.473389 | 0.337776 | -1.15259 | 1.49036954 | ANN |
| *Gj2A255T25* | 0 | 4.739927 | 4.704738 | 4.877111 | 4.841923 | 0.03518891 | ANN |
| *Gj2A256T37* | -0.17638 | -0.16222 | -0.6303 | 0.014163 | -0.45392 | 0.46808274 | ANN |
| *Gj2A256T38* | -0.84115 | -0.36487 | -3.93156 | 0.476283 | -3.09041 | 3.56669553 | ANN |
| *Gj2P256T7* | -1.3515 | -0.54828 | -4.11963 | 0.803218 | -2.76812 | 3.57134114 | ANN |
| *Gj8A380T71* | 2.090166 | 1.978264 | 0.819141 | -0.1119 | -1.27103 | 1.15912317 | ANN |
| *Gj9A43T71* | 0.992846 | 0.453855 | 1.909498 | -0.53899 | 0.916652 | -1.455643 | ANN |
| *Gj8A5T50* | -0.38887 | -0.27491 | -0.48422 | 0.113958 | -0.09536 | 0.20931445 | GI |
| *Gj8P5T9* | -0.40909 | -0.38027 | -0.59992 | 0.028815 | -0.19083 | 0.21964498 | GI |
| *Gj9A812T105* | 0.102645 | 0.114055 | 0.150372 | 0.01141 | 0.047727 | -0.0363164 | H_PPase |
| *Gj4A0T68* | 1.443499 | 0.91284 | 1.020459 | -0.53066 | -0.42304 | -0.1076191 | PKS |
| *Gj5A312T50* | 0.351845 | 0.122019 | 0.070125 | -0.22983 | -0.28172 | 0.0518941 | PKS |
| *Gj11A261T51* | -0.45993 | -0.31642 | -0.12765 | 0.143513 | 0.332284 | -0.1887707 | SOS1 |
| *Gj9A34T69* | -0.1474 | -0.01807 | -0.41154 | 0.12933 | -0.26413 | 0.39346294 | SOS2 |
| *Gj10A292T112* | 0.474832 | 0.282971 | 0.495734 | -0.19186 | 0.020902 | -0.212763 | V-ATPase |
| *Gj1A518T64* | 0.545588 | 0.234423 | 0.711986 | -0.31116 | 0.166398 | -0.477563 | V-ATPase |
| *Gj1P423T5* | 0.065093 | 0.094396 | 0.375633 | 0.029302 | 0.31054 | -0.2812378 | V-ATPase |
| *Gj7A89T20* | 0.09632 | 0.121318 | 0.652419 | 0.024998 | 0.556099 | -0.5311012 | V-ATPase |
| *Gj7X223T16* | -0.04988 | 0.535096 | -0.41186 | 0.584972 | -0.36199 | 0.94695748 | V-ATPase |
| *Gj8A273T9* | -0.69675 | -0.13405 | -0.15406 | 0.562701 | 0.54269 | 0.02001063 | V-ATPase |
| *Gj8A274T18* | 0.102601 | 0.018084 | 0.210912 | -0.08452 | 0.108311 | -0.1928279 | V-ATPase |
| *Gj8A280T37* | 0.467683 | 0.390919 | 0.459157 | -0.07676 | -0.00853 | -0.0682377 | V-ATPase |
| *Gj9A1013T95* | 0.479315 | 0.182117 | 1.163949 | -0.2972 | 0.684634 | -0.9818316 | V-ATPase |
| *Gj9A231T88* | -0.34137 | -0.62875 | -0.20419 | -0.28738 | 0.137179 | -0.4245542 | V-ATPase |
| *Gj9A820T119* | -0.31839 | -0.55238 | -0.05131 | -0.23399 | 0.267075 | -0.5010661 | V-ATPase |
| *Gj9A968T97* | 0.639641 | 0.515739 | 0.767091 | -0.1239 | 0.12745 | -0.2513525 | V-ATPase |
| *Gj9A968T98* | 0.348481 | 0.108243 | 0.39374 | -0.24024 | 0.045258 | -0.2854968 | V-ATPase |
| *Gj9P867T21* | 0.977084 | 1.286838 | 1.783329 | 0.309754 | 0.806245 | -0.4964911 | V-ATPase |
| *Gj9P985T6* | 0.539557 | -0.10046 | -0.3421 | -0.64001 | -0.88166 | 0.24164065 | V-ATPase |
